# Photosystem II‐Carbon Nitride Photoanodes for Scalable Biophotoelectrochemistry

**DOI:** 10.1002/adma.202508813

**Published:** 2025-08-15

**Authors:** Huayang Zhang, Wenjie Tian, Jingkai Lin, Peng Zhang, Guosheng Shao, Sai Kishore Ravi, Hongqi Sun, Emiliano Cortés, Virgil Andrei, Shaobin Wang

**Affiliations:** ^1^ School of Chemical Engineering The University of Adelaide Adelaide SA 5005 Australia; ^2^ Nanoinstitute Munich Faculty of Physics Ludwig‐Maximilians‐Universität München 80539 Munich Germany; ^3^ School of Materials Science and Engineering Zhengzhou University Zhengzhou, Henan 450001 China; ^4^ School of Energy and Environment City University of Hong Kong Kowloon 999077 Hong Kong; ^5^ School of Molecular Sciences The University of Western Australia Perth WA 6009 Australia; ^6^ School of Materials Science and Engineering Nanyang Technological University Nanyang 639798 Singapore; ^7^ Yusuf Hamied Department of Chemistry University of Cambridge Cambridge CB2 1EW UK

**Keywords:** artificial photosynthesis, biophotovoltaic, macroporous carbon nitride, photoelectrochemistry, photosystem II

## Abstract

Photosystem II (PSII) is a vital photosynthetic enzyme with the potential for sustainable bioelectricity and fuel generation. However, interfacing PSII with intricate, small‐scale electrodes for practical applications has been challenging. This study addresses this by creating protonated macroporous carbon nitride (MCN) as support and developing a scalable spray‐freeze method to wire PSII with MCN. This facilitates the production of large‐area MCN‐PSII photoanodes up to 33 cm^2^ for biophotoelectrochemical water oxidation to O_2_, achieving efficient interfacial charge transfer and initial photocurrents in the mA range with Faradaic yield of 93.5 ± 8.5% over 5 h. A bias‐free biophotoelectrochemical (BPEC) device is designed by connecting the MCN‐PSII photoanode to a carbon nanotube cathode loaded with bilirubin oxidase. An array of eight tandem BPEC cells with a photoactive area of 72 cm^2^ successfully powers low‐power electronics, such as LEDs. This work paves an efficient way for bioelectrode fabrication, showcasing the potential of PSII‐based semi‐artificial systems for BPEC and biophotovoltaic applications.

## Graphical abstract

A spray‐freeze method is developed to fabricate large‐area (up to 33 cm^2^) macroporous carbon nitride photoanodes integrated with photosystem II enzymes for biophotoelectrochemical systems. The photoanodes reach a Faradaic efficiency of 93.5% for O_2_ evolution. Bias‐free tandem biophotovoltaic devices with a total photoactive area of 72 cm^2^ drive low‐power electronics, highlighting the promise of PSII‐based platforms for sustainable energy conversion.

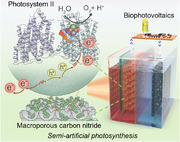

## 1 Introduction

Photosystem II (PSII, Figure S1s Information) enzymes, known as the only water oxidase in nature, exhibit remarkable efficiency and selectivity in photosynthesis, evolving O_2_ at a high rate.^[^ ^1^ ^]^ This process also generates protons and electrons for the fixation of carbon dioxide into sugars (Figure S2a, Supporting Information),^[^ ^2^ ^]^ facilitating biomass production.^[^ ^3^ ^]^ While PSII organisms are abundant and regenerable in the form of inexpensive raw biomass, they cannot be used directly in the biological industry due to their complex metabolic pathways.^[^ ^4^ ^]^ In contrast, artificial photosynthesis, such as photoelectrochemical (PEC) water splitting (Figure S2b, Supporting Information), offers technical flexibility and ease of handling for solar energy conversion.^[^ ^5^ ^]^ However, the sluggish kinetics of water oxidation by synthetic, mostly inorganic (photo)electrocatalysts hinders overall PEC performance.^[^ ^6^ ^]^

To bridge the gap between the high activity of natural enzymes and the versatility of artificial materials, researchers have explored the integration of isolated PSIIenzymes with artificial electrode materials (Figure S2c, Supporting Information).^[^ ^7^, ^8^ ^]^ This biotic/abiotic setup combines the advantages of photosynthetic machinery with PEC systems, enabling a controlled and sustainable semi‐artificial configuration for photosynthesis, light‐to‐electricity conversion, or biosensing applications.^[^ ^9^, ^10^, ^11^ ^]^ Previous studies have employed Au,^[^ ^12^ ^]^ graphene,^[^ ^13^ ^]^ carbon nanotubes^[^ ^14^ ^]^ and porous carbon,^[^ ^15^ ^]^ or metal oxide semiconductors including TiO_2_,^[^ ^8^ ^]^ Fe_2_O_3_,^[^ ^16^ ^]^ and indium tin oxide (ITO)^[^ ^17^ ^]^ to immobilize PSII dimers. However, conventional drop‐casting, electrodeposition, or spin‐coating methods limit the scalability of inorganic^[^ ^18^, ^19^ ^]^ and PSII^[^ ^8^, ^14^ ^]^ photoanodes to active areas <1 cm^2^, which prevents extensive studies on their real‐world applicability. While spray coating or spray pyrolysis emerge as alternatives for inorganic photoelectrode deposition,^[^ ^20^, ^21^ ^]^ these often require heat processing (e.g., 170 °C, 450 °C)^[^ ^22^, ^23^ ^]^ to enhance interfacial bonding strength and improve charge transfer/separation. However, this heat treatment is not compatible with enzymes, as PSII deactivation is observed above 45 °C.^[^ ^24^ ^]^ Scaling up PSII electrodes from small to large sizes presents significant challenges, particularly in maintaining strong adhesion and uniform attachment of PSII across the entire substrate. Additionally, as the electrode size increases, elevated resistive losses make it difficult to sustain a high photocurrent output. Possibly due to these challenges, a single 25 cm^2^ ITO/PSII electrode has been reported,^[^ ^25^ ^]^ but its photocurrent response and biointerface have not been investigated.

**Figure 1:**
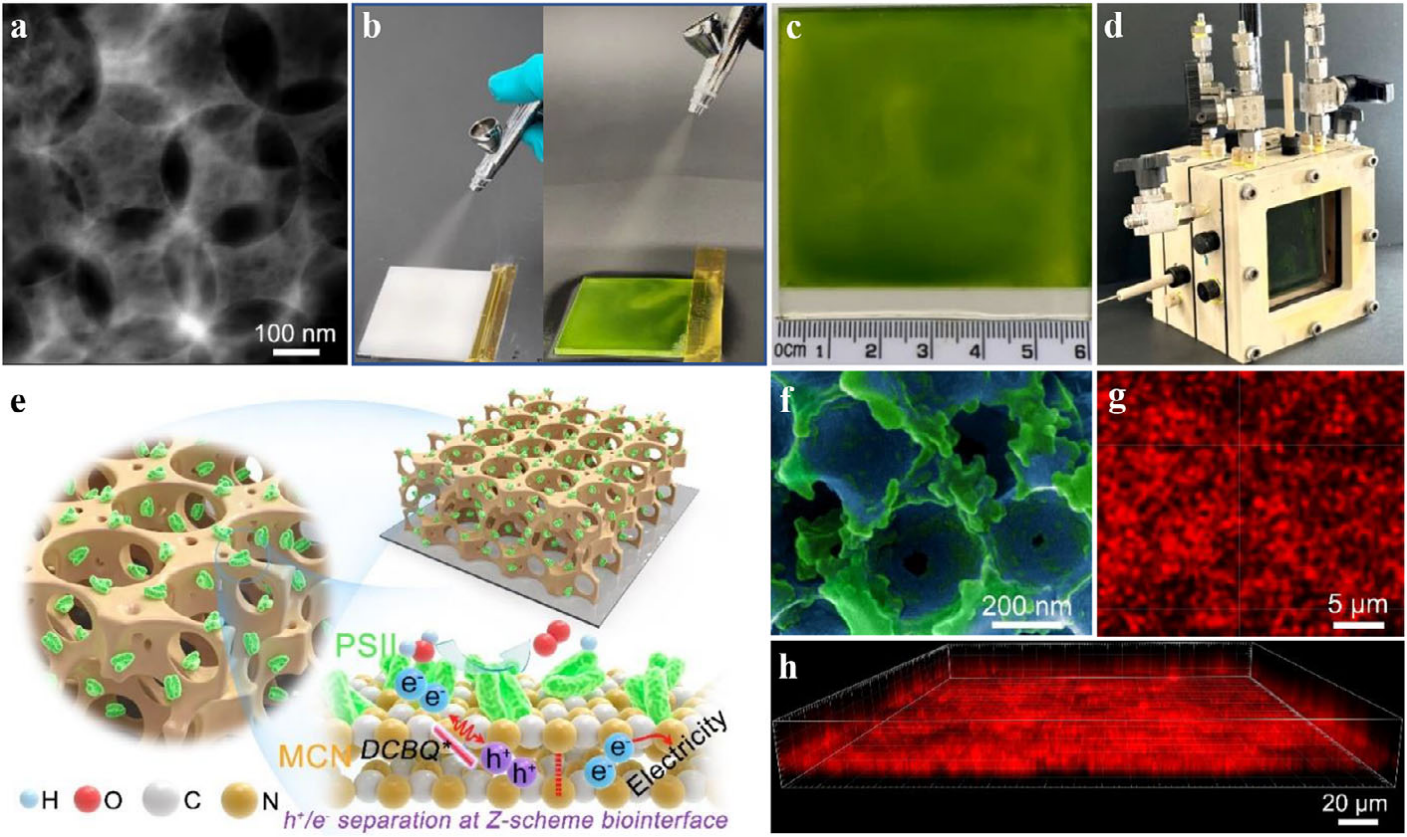
Fabrication and characterization of the MCN‐PSII photoanode. a) HAADF‐STEM image of MCN. b) Scalable MCN‐PSII photoanode fabrication steps: MCN spraying on FTO glass (left), followed by PSII spraying on MCN electrode (right). After PSII incubation, samples are freeze‐treated at ‒80 °C (see Experimental Section). c) Large‐scale, 33 cm^2^ MCN‐PSII photoanode. d) Set up for large‐scale PEC tests. e) Schematic diagram of the anchoring of PSII proteins on MCN and the electron transfer pathway on the biointerface. f) Colorized SEM image. g) CLSM image (excited at 559 nm). h) Spatial PSII distribution on MCN‐PSII electrodes.

In this work, we aim to synthesize PSII electrodes from <1 to >30 cm^2^ for biophotoelectrochemical (BPEC) solar energy conversion, with the goal of maintaining an effective biointerface and consistent photocurrent output even after electrode scaling. The protonated macroporous carbon nitride (MCN, **Figure** **1**a) is developed as an innovative support material for PSII. Carbon nitride offers multiple advantages over established electrode materials like ITO,^[^ ^17^, ^25^ ^]^ as it is non‐toxic, metal‐free, chemically stable, and cost‐effective.^[^ ^26^, ^27^ ^]^ Furthermore, MCN exhibits auxiliary visible light absorption, enhancing the light‐harvesting capability of the PSII photoanode, while the robust Z‐scheme MCN‐PSII biointerface allows for effective charge separation. In comparison, while ITO substrates benefit from transparency and conductivity, they lack PEC activity, contributing little to the overall performance of the PSII photoanode.^[^ ^28^ ^]^

To make use of the advantages of MCN and ensure biointerface stability and adhesion when scaling up PSII electrodes, we introduce a spray‐freeze technique that enables the facile deposition of 33 cm^2^ MCN‐PSII photoanodes, demonstrating large‐scale BPEC O_2_ evolution. The activity of PSII on a 33 cm^2^ planar bulk carbon nitride (b‐CN) electrode was also studied for comparison. Our research shows that spray coating provides a uniform deposition of PSII films, whereas freeze treatment enhances the biotic/abiotic interaction between PSII and MCN or b‐CN, leading to better charge transfer and separation. When fabricating O_2_‐reducing bilirubin oxidase (BOD) modified carbon nanotube (CNT) film cathode, the freeze treatment also improves the interfacial electrical communication for better catalytic kinetics in electrocatalytic oxygen reduction reaction (ORR). This highlights the potential of the spray‐freeze method as a general and scalable strategy for fabricating electrodes with enhanced interfacial integration. A bias‐free tandem BPEC device is further demonstrated by connecting the MCN‐PSII photoanode with a BOD‐modified CNT film cathode. Although the power densities we observed are lower compared to those obtained in artificial solar‐to‐electricity or solar‐to‐fuel systems,^[^ ^29^ ^]^ our study indicates that functional PSII‐based photoanodes could be scaled up while retaining effective and sustainable photoelectric output. Eight serially connected tandem BPEC devices can supply low‐power LED electronics, paving the way for the practical implementation of PSII in BPEC and biophotovoltaic (BPV) systems.

## 2 Results and Discussion

### 2.1 Synthesis of PSII‐Based Photoanodes

A spray‐freeze technique was developed to obtain a uniform and close biointerface for large‐scale photoanodes. To this end, MCN, b‐CN, and PSII suspensions were sprayed onto conductive fluorine‐doped tin oxide (FTO) glass using a commercial airbrush (Figure 1b; Figure S3a, Supporting Information), followed by freeze treatment (see Experimental Section). The resulting materials and electrodes were characterized by high‐angle annular dark‐field scanning transmission electron microscopy (HAADF‐STEM), scanning electron microscopy (SEM), X‐ray photoelectron spectroscopy (XPS), X‐ray powder diffraction (XRD), Fourier‐transform infrared spectroscopy (FT‐IR), and confocal laser scanning microscopy (CLSM). MCN possesses ordered macropores with a diameter of ≈300 nm (Figure 1a; Figures S4 and S5, Supporting Information), and interconnected pores enabling PSII access and retention (10.5 × 11 × 20.5 nm for a PSII dimer).^[^ ^30^ ^]^ In contrast, b‐CN exhibits a bulk and planar morphology (Figure S6, Supporting Information), restricting PSII loading to the outer surface rather than within the structure. XRD patterns of MCN and b‐CN reveal two characteristic (100) and (002) diffraction peaks (Figure S7, Supporting Information). FT‐IR (Figure S8, Supporting Information) and XPS with or without in situ irradiation (Figure S9, Supporting Information) were conducted for structural analysis of planar b‐CN and MCN. Compared to b‐CN with no spectrum changes, N 1s spectrum of MCN exposed a slight positive shift (Figure S8c, Supporting Information) under irradiation, suggesting an electrostatic shielding effect and improved photoelectron transfer.^[^ ^31^ ^]^ The weak peak at 404.1 eV (Figure S8d, Supporting Information) indicates the protonation of MCN by NH_4_HF_2_ etching during preparation, which makes heterocycles more positively charged. ^1^H nuclear magnetic resonance (NMR) spectra and Zeta potential changes further proved the protonation of MCN, as analyzed in Figure S10 (Supporting Information). Similar protonation behavior has been reported for mesoporous‑C_3_N_4_, which exhibits an apparent p*K*_a_ = 6.60  ±  0.3.^[^ ^32^ ^]^ Time‐resolved transient photoluminescence (PL) and steady‐state PL spectra (Figure S11, Supporting Information) supported the improved charge transfer and separation of MCN compared to b‐CN. To quantitatively assess this difference, we performed biexponential fitting on the decay profiles (Figure S12a, Supporting Information). The results show that, compared to bulk b‐CN, MCN exhibits a shorter fast decay lifetime (τ₁) and a slightly reduced average lifetime (τ_avg_), indicating more efficient charge separation and transfer pathways. MCN electrodes of 0.196 cm^2^ active areas showed better film formation on FTO glass substrates than b‐CN prepared via drop‐casting (Figure S13a,c and S14, Supporting Information). MCN‐PSII and b‐CN‐PSII electrodes of 0.196 cm^2^ (Figure S14b,d, Supporting Information) were prepared by self‐assembly of PSII via dip coating. Compared to b‐CN, the ordered macroporous channels and protonated sites on MCN will facilitate the spatial interaction with negatively charged PSII (Figure 1e; Figure S12b, Supporting Information). Alternatively, 3 × 3 (9) cm^2^ and 6 × 5.5 (33) cm^2^ electrodes were prepared using the spray‐freeze approach (Experimental Section). As shown in Figure 1b, Figures S15a,c and S16a (Supporting Information), spray‐coated MCN and b‐CN show better uniformity than drop‐cast MCN and b‐CN. Spraying also ensures a uniform loading of the PSII film on MCN or b‐CN (Figure 1c; Figures S15b and S16b, Supporting Information), as PSII solution drop‐cast on the 33 cm^2^ electrode surface does not spread evenly due to surface tension effects (Figure S17, Supporting Information). These results suggest that when using the spray‐freeze method, additional protonation treatment is not essential for achieving effective PSII immobilization. SEM images of MCN‐PSII (Figure 1f; Figure S18, Supporting Information) indicate isolated and agglomerated PSII monomers attached to the macropore walls. Based on the fluorescence emission from chlorophyll *a* (Chl *a*), CLSM further confirmed the 3D penetration of PSII dimers throughout the porous MCN layer (Figure 1g,h; Figures S19 and S20a, Supporting Information). In contrast, PSII are aggregated and stacked along the upper b‐CN surface (Figures S20b and S21, Supporting Information), forming a 2D biointerface.

**Figure 2:**
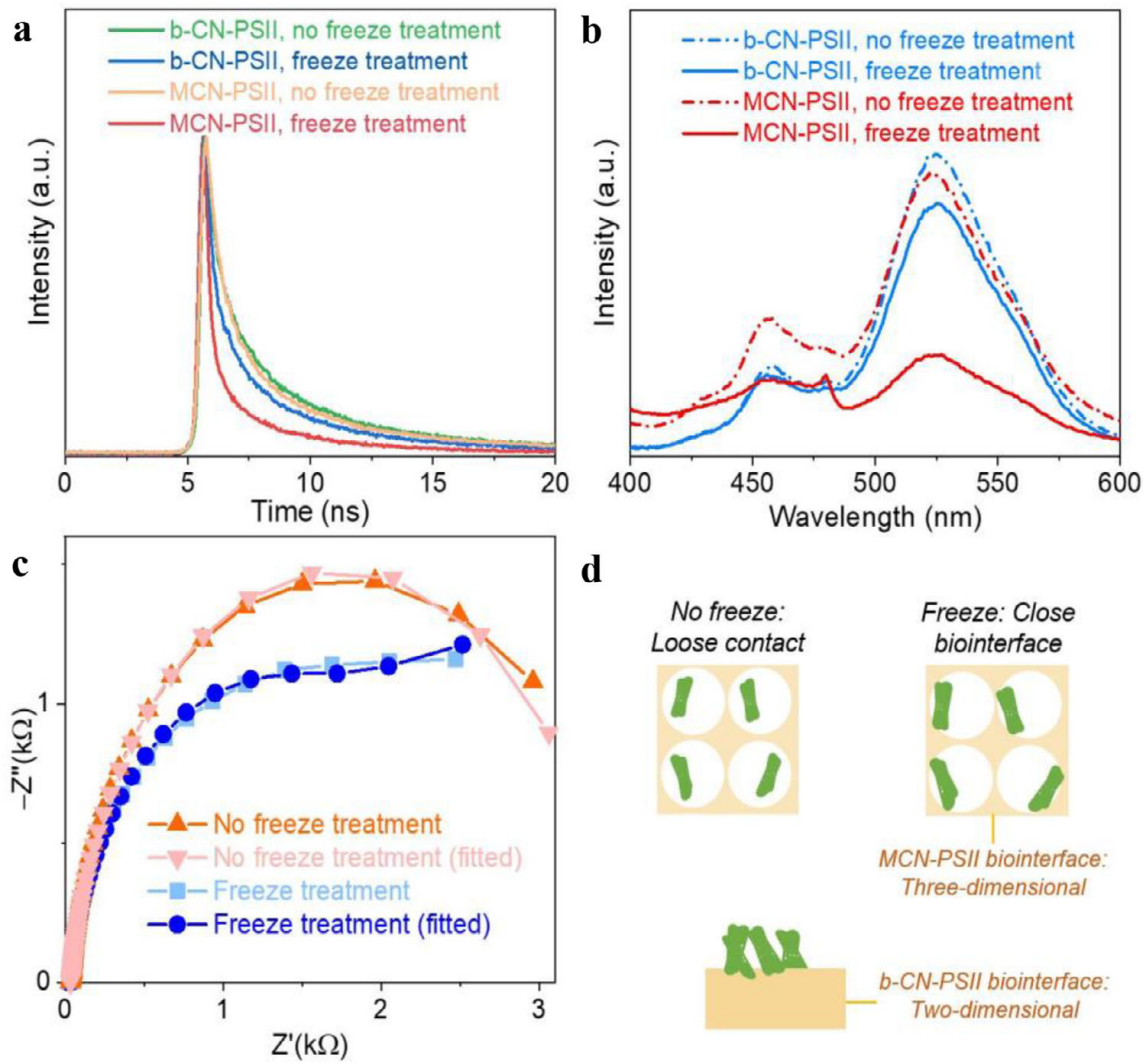
The effect of freeze treatment on 9 cm^2^ MCN‐PSII and b‐CN‐PSII electrodes. a) Transient PL and b) steady‐state PL spectra for electrodes with or without freeze treatment. c) MET EIS spectra under light irradiation of MCN‐PSII, fitted based on Figure S27 (Supporting Information). d) Schematic illustration of the close MCN/PSII biointerface obtained during freeze treatment for MCN‐PSII and b‐CN‐PSII electrodes.

While spraying (Figure 1b) ensures a uniform film deposition, the subsequent freeze treatment (Experimental Section) is key to attaining a close abiotic/biotic interface for spray‐coated MCN‐PSII or b‐CN‐PSII electrodes. SEM images show little difference (Figure S22, Supporting Information), while TEM images (Figure S23, Supporting Information) indicate that MCN particles are enveloped in a PSII film before treatment, whereas PSII is better distributed within the MCN macropores after freeze treatment, suggesting that PSII interacts more closely with MCN. Transient and steady‐state PL spectra in **Figure** **2**a,b confirm that freeze treatment can improve charge transfer and separation in MCN‐PSII and b‐CN‐PSII. TRPL results (Figure S12b,c, Supporting Information) reveal evident decreases in average fluorescence lifetime after freeze treatment (τ_avg_ = 2.19 → 1.63 ns for b‐CN‐PSII; τ_avg_ = 2.08 → 0.94 ns for MCN‐PSII), consistent with faster exciton dissociation and more efficient interfacial electron transfer. Moreover, LSV curves and Tafel plots reveal marginally reduced Tafel slopes after freeze treatment, indicating slightly enhanced reaction kinetics (Figures S24a–c and S25a,b, Supporting Information). Electrochemical impedance spectra (EIS) further indicate a decreased interfacial charge transfer resistance in MCN‐PSII and b‐CN‐PSII after freeze treatment (Figure 2c; Figures S25c,d and S26, Supporting Information). These results suggest that a closer enzyme‐MCN surface interaction may occur during the freezing process, which is maintained after thawing (Figure 2d). Freeze treatment has a more critical effect on MCN‐PSII with a 3D electrode structure, in comparison to b‐CN‐PSII with a 2D interface contact, as the interconnected macroporous network increased the surface area available for interactions with PSII (Figure 2d). Additionally, a comparative study was performed on MCN electrodes. EIS, and LSV results (Figure S24d,e, Supporting Information) reveal that freeze treatment improves the contact between MCN and the FTO substrate, leading to reduced charge transfer resistance, enhanced charge transfer efficiency, and improved PEC performance.

### 2.2 Photoanode Characterization

**Figure 3:**
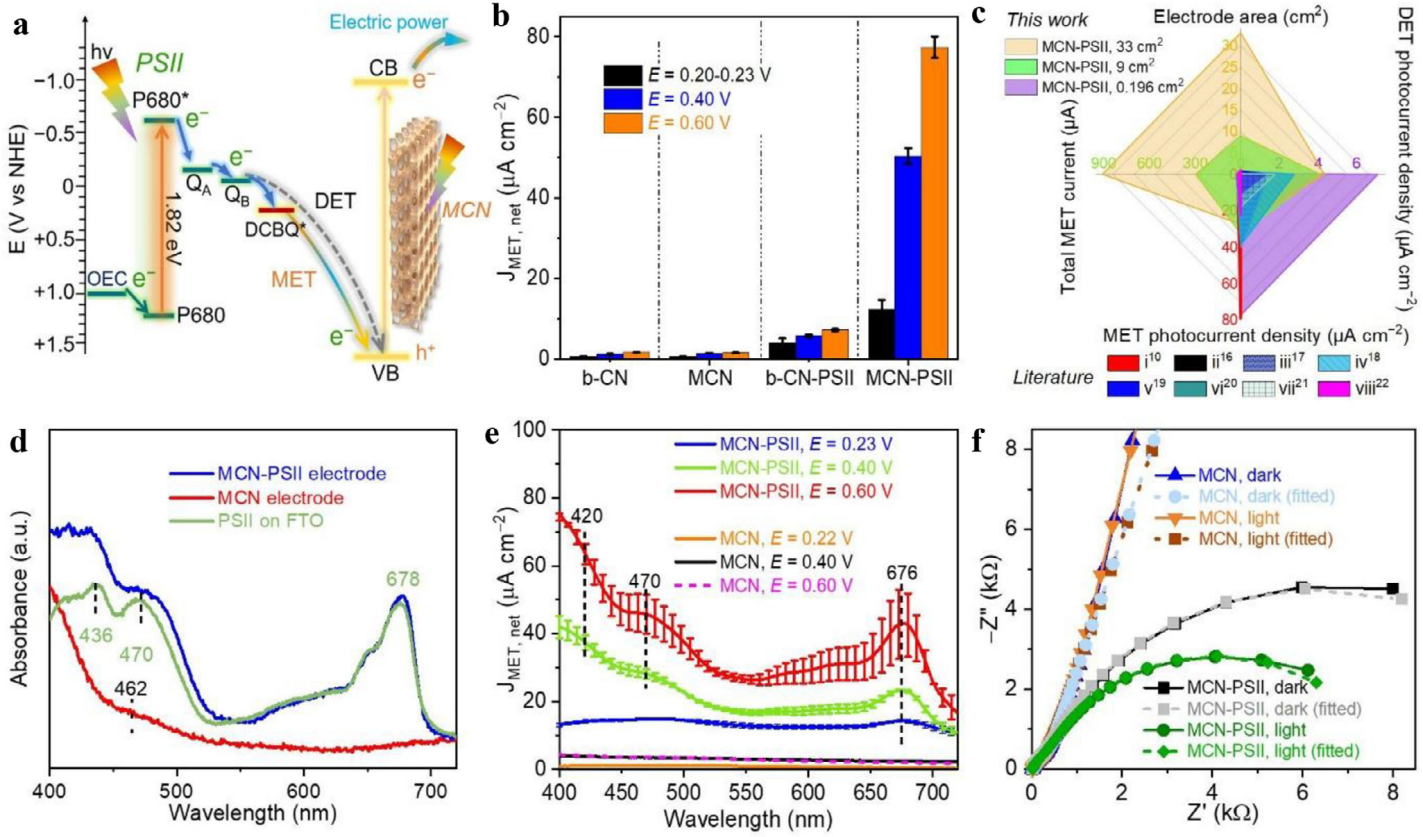
Energy level diagram, optical and PEC characterization of 0.196 cm^2^ photoanodes under air. a) Energy level diagram of the cascade DET or MET processes on MCN‐PSII electrodes. b) Average J_MET, net_ values at different E versus SCE based on results in Figures S31 and S32 (Supporting Information). c) Performance comparison between MCN‐PSII electrodes and literature reports (labels correspond to reference numbers from Table S2 (Supporting Information), with different PSII electrode configurations: i) IO‐TiO_2_|dpp|P_Os_‐PSII; ii) ITO‐(PEI‐rGO/PSII)_3_; iii) PSII on ITO‐SA 750 nm; iv) PSII/(ITO)_15_/Au; v) PSII/(TiO_2_)_5_‐NTs/ITO; vi) PSII/NCNT; vii) C‐PEI‐PSII; viii) PSII‐modified ITO. d) UV‐vis diffuse reflectance spectra of MCN, PSII, and MCN‐PSII electrodes (spectra of b‐CN and b‐CN‐PSII electrodes are provided in Figure S39, Supporting Information). MCN provides auxiliary light adsorption to PSII at wavelengths<462 nm (absorption edge of MCN). e) MET photocurrent action spectra at different E versus SCE and under continuous monochromatic light irradiation (400–720 nm, light intensity is shown in Figure S40, Supporting Information). The spectra depict J_MET, net_ after subtracting the corresponding dark currents (Figures S41 and S42, Supporting Information). Photocurrent peaks at 676 and 470 nm arise from the excitation of PSII *Q_y_* band and *β‐*carotene, respectively, whereas the photocurrent at λ≤420 nm stems from the excitation of PSII *B_x_* and *B_y_* bands.^[^ ^8^ ^]^ f) MET EIS of MCN and MCN‐PSII electrodes obtained under dark or light irradiation, fitted based on Figure S27 (Supporting Information).

To gain deeper insight into the electronic structure and PEC behavior of the fabricated photoanodes, a series of spectroscopic and PEC characterizations were conducted on 0.196 cm^2^ photoanodes. The band structure analysis of MCN, b‐CN, including UV‐Vis diffuse reflectance spectra, transformed Kubelka‐Munk functions versus photon energy, Mott‐Schottky plots, and energy level diagrams of MCN‐PSII, b‐CN‐PSII photoanodes (**Figure** **3**a; Figures S28 and S29, Supporting Information) revealed the formation of MCN‐PSII or b‐CN‐PSII Z‐scheme structures (illustrated in Figure S29, Supporting Information). The improved charge separation at the biointerface endows PSII with a higher water oxidation efficiency and enhanced electron transfer from the conduction band (CB) of MCN to the counter electrode via an external circuit, generating an anodic direct electron transfer (DET) photocurrent.^[^ ^33^, ^34^ ^]^ Electron transfer in DET process can only be realized when the electroactive sites of PSII face the bio‐interface. As a diffusional electron transfer mediator, 2,6‐dichloro‐1,4‐benzoquinone (DCBQ) can be introduced to accelerate electron transfer from Q_B_ of PSII to MCN (Figure 1e),^[^ ^35^ ^]^ producing a mediated electron transfer (MET) photocurrent. DCBQ enables PSII to relay electrons to the electrode, regardless of its orientation, so most photo‐generated electrons in Q_B_ can be conveyed to the electrodes, demonstrating notably improved photo‐response.

Based on linear sweep voltammetry (LSV, Figure S30, Supporting Information), initially detected open circuit potential values, 0.40 and 0.60 V versus saturated Hg/Hg_2_Cl_2_ electrode (SCE), were selected as the applied potential (E) during PEC tests of 0.196 cm^2^ electrodes. Chopped‐light chronoamperometry further indicated an optimal electrode thickness of 28 µm (Figure S31, Supporting Information). J_DET, net_ and J_MET, net_ were defined as the absolute DET, and MET photocurrent density (J), respectively, obtained after subtracting the dark current density.^[^ ^8^ ^]^ b‐CN and MCN electrodes showed weak photoresponses, while b‐CN‐PSII and MCN‐PSII photoanodes exhibited improved J_DET, net_ and J_MET, net_ (Figure 3b; Figures S32–S34 and Table S1, Supporting Information). This verified the effective electronic communication between PSII and MCN or b‐CN^[^ ^16^ ^]^ for anodic photocurrent generation and the decisive contribution of PSII to PEC water oxidation. MCN‐PSII delivered the highest J_DET, net_ (7.1 ± 1.0 µA cm^−2^), and J_MET, net_ (77.4 ± 2.6 µA cm^−2^, over 10 times higher than that of b‐CN‐PSII) at 0.60 V versus SCE. The amounts of Chl *a* on b‐CN‐PSII and MCN‐PSII electrodes were determined to be 0.9 ± 0.2 and 2.6 ± 0.4 µg (Table S1 and Figure S35, Supporting Information), respectively. This illustrates that MCN improves the immobilization of PSII dimers in 0.196 cm^2^ electrode. The J_DET, net_ and J_MET, net_ of 0.196 cm^2^ MCN‐PSII are within the range reported for graphene, CNT, porous carbon, or metal oxide supported PSII electrodes (Figure 3c; Table S2, Supporting Information), with further optimization needed to enhance photocurrent densities.

Under these conditions, 33 cm^2^ MCN‐PSII electrodes attained net MET photocurrent (I_MET, net_) of 907.8 µA at 0.60 V versus SCE, attaining unprecedented scalability (Figure 3c; see PEC performance of 9 and 33 cm^2^ MCN‐PSII and MCN photoanodes in Figures S36–S38 and Table S1, Supporting Information). The J_MET, net_ of 9 and 33 cm^2^ MCN‐PSII electrodes (Table S1, Supporting Information) was lower compared to small‐scale (0.196 cm^2^) electrodes. This arises from an increased sheet resistance over larger electrode areas, which limits the overall photocurrent.

The UV‐vis spectrum of MCN electrodes (Figure 3d) shows visible light absorption, with an absorption edge of 462 nm, while typical Chl *a* absorption peaks of 432, 483, and 678 nm are observed for MCN‐PSII electrodes. MCN samples showed negligible monochromatic DET (Figure S41c, Supporting Information) and MET photocurrents (Figure 3e). In contrast, MCN‐PSII electrodes display significantly higher J_DET, net_ (Figure S42a, Supporting Information) and J_MET, net_ (Figure 3e) values in the 400–720 nm range, proving the major contribution of PSII to the anodic photocurrent. Incident photon‐to‐current efficiencies (IPCE) spectra (Figure S43b,c, Supporting Information) indicated that MCN‐PSII photoanodes could perform photon‐to‐current conversion over a broad visible region (λ >400 nm), consistent with its UV–vis spectrum. MET IPCE values of MCN‐PSII samples peaked at ≈400 nm (8.5 ± 0.1%) and 670 nm (2.9 ± 0.7%) at 0.60 V versus SCE, which are 17 and 26 times higher than IPCE values observed in the DET process. This performance is lower than previous reports of PSII on dye‐sensitized inverse opal TiO_2_^[^ ^8^ ^]^ but exceeds porous carbon‐supported PSII electrodes.^[^ ^15^ ^]^ Monochromatic photocurrents and IPCE values of MCN‐PSII photoanodes further surpassed those of b‐CN‐PSII photoanodes (Figures S44 and S45, Supporting Information). EIS in Figure 3f depicts a lower resistance of MCN‐PSII electrodes in the low‐frequency region over MCN. This indicates a reduced photo‐induced electron transfer resistance and accelerated surface kinetics for the constructed Z‐scheme biointerface, which ensures efficient interfacial charge transport.

### 2.3 Large‐Scale PEC O_2_ Evolution

**Figure 4:**
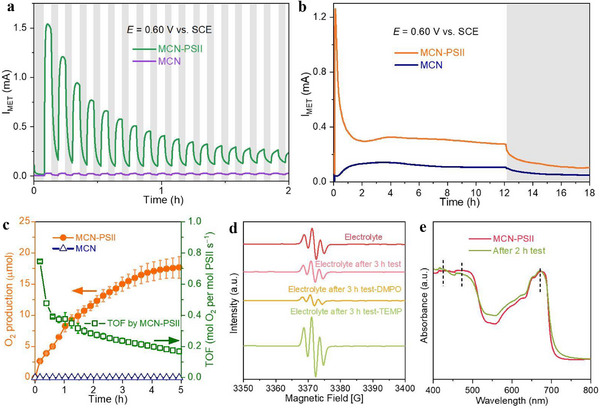
PEC performance of large‐area electrodes under N_2_ atmosphere. a) MET photocurrents of 33 cm^2^ MCN‐PSII and MCN photoanodes over 200 s on‐off light cycles. b) MET photocurrents of 33 cm^2^ MCN‐PSII and MCN photoanodes over 12 h light/6 h dark (AM 1.5 G, 80 mW cm^2^). Grey areas indicate dark periods. c) O_2_ evolution and corresponding TOF of MCN‐PSII and MCN photoanodes during the first 5 h of irradiation in (b) and Figure S48a (Supporting Information). d) Electron paramagnetic resonance (EPR) spectra of the electrolyte before and after 3 h MET PEC tests on 33 cm^2^ MCN‐PSII (Figure S51, Supporting Information). 2,2,6,6‐Tetramethylpiperidine (TEMP) was used for capturing singlet oxygen (^1^O_2_). 5,5‐Dimethyl‐1‐pyrroline N‐oxide (DMPO) was adopted for hydroxyl radical (•OH) detection. Only DCBQ peaks emerged when either TEMP or DMPO were used, indicating that no •OH and ^1^O_2_ species were produced in the electrolyte during the PEC test. e) UV‐vis spectra of 33 cm^2^ MCN‐PSII electrodes before and after 2 h PEC tests, recorded on the same electrode.

Next, we investigated the activity of 33 cm^2^ MCN and MCN‐PSII photoanodes toward O_2_ evolution in a 3‐electrode setup at 0.60 V versus SCE, under inert N_2_ atmosphere (see Experimental Section). The 3‐electrode setup employed a Pt counter electrode producing H_2_ on the cathodic side (see example in Figure S46, Supporting Information). MCN‐PSII produced an initial MET photocurrent of 1.33 ± 0.16 mA during chopped light cycles (200 s on‐off intervals; **Figure** **4**a; Figure S47, Supporting Information), which decayed to 0.26 ± 0.01 mA after 2 h and 0.22 ± 0.01 mA after 12 h. In contrast, the MCN photoanode exhibited a low MET photocurrent of ≈0.016–0.032 mA over a 12 h test.

We further probed the MET photocurrent under 12 h light/6 h dark cycles (Figure 4b; Figure S48a, Supporting Information). In this case, the photocurrent of an MCN electrode increased over the first 2 h, which may be due to slow charge transfer kinetics as hinted in Figure 3f. In contrast, the MCN‐PSII electrode demonstrated fast electron transfer kinetics in both 200 s/200 s and 12 h/6 h chopped light tests and a similar photocurrent decay over the first 2 h. This corresponded to a photocurrent half‐life time (τ^1/2^) of ≈23 min, which is consistent with previous reports of immobilized PSII photoanodes.^[^ ^8^, ^17^, ^36^ ^]^ The decay may be caused by PSII photodamage that occurs as light irradiation impairs the Mn_4_Ca catalytic site of the oxygen‐evolving complex (OEC), with additional effects on the quinone electron acceptors and tyrosine donors of PSII.^[^ ^37^, ^38^ ^]^ Long‐term PEC tests revealed a much slower decay of MCN‐PSII photocurrents beyond the first 2 h. While MCN‐PSII photoanodes sustained higher MET photocurrents than MCN electrodes over 96 h tests (Figure S48a, Supporting Information), the half‐life time indicates that this residual long‐term activity may originate from the presence of other species including PSII degradation products.^[^ ^39^ ^]^ Accordingly, UV‐vis spectra of MCN‐PSII electrodes (Figure 4e) indicated no significant changes in the characteristic absorption peaks of Chl *a* after 2 h of irradiation, whereas lower Chl *a* absorption peaks were retained on MCN‐PSII after 96 h (Figure 4e; Figure S48b, Supporting Information).

The produced O_2_ amounted to 17.8 ± 1.8 µmol O_2_ after 5 h irradiation (Figure 4c), which corresponds to a Faradaic yield of 93.5 ± 8.5%. Beyond 5 h, the O_2_ evolution reached a plateau, and the signal became less reliable due to both declining PSII activity and instrumental limitations in an open‐flow setup with carrier gas and mass flow control (as detailed in the Experimental section). Accordingly, the turnover frequency (TOF) of the 33 cm^2^ MCN‐PSII electrode decreased from 0.75 to 0.17 s^−1^ (Figure 4c), whereas no O_2_ was quantified for the MCN system. Only negligible amounts of H_2_O_2_ were observed after long‐term PEC tests (Figure S50, Supporting Information), whereas no reactive •OH, and ^1^O_2_ species were detected in the electrolyte of MCN‐PSII (Figure S51, Supporting Information) and b‐CN‐PSII electrodes (Figure S52a, Supporting Information) by EPR (Figure 4d; Figure S53, Supporting Information).

To rationalize this photocurrent decay, we performed a series of subsequent control tests. Front irradiation revealed a similar photocurrent behavior and marginally decreased performance over back irradiation, which may be due to the accelerated photodegradation of PSII^[^ ^37^, ^38^ ^]^ by direct exposure to irradiation (Figure S49b, Supporting Information). N_2_ purging did not have a significant effect on the MCN‐PSII photoresponse compared to electrolytes exposed to air. A higher photocurrent could be regained after 24 h by replacing the electrolyte with a fresh solution with 1 mm DCBQ (Figure S51, Supporting Information), which indicates that DCBQ degradation also contributes to the gradual decrease in performance. This DCBQ degradation could be traced by EPR spectra (Figure S53, Supporting Information). Subsequent addition of 3‐(3,4‐dichlorophenyl)‐1,1‐dimethylurea (DCMU), an inhibitor that binds to Q_B_ in PSII,^[^ ^40^ ^]^ resulted in a decrease of I_MET_ (Figures S51 and S52a, Supporting Information). However, the dark current also declined to some extent, showing no PSII‐specific effect that would validate its long‐term activity. PSII was not detected in the electrolyte solutions of MCN‐PSII after 4 days or b‐CN‐PSII after 6.6 days (Figure S54, Supporting Information), indicating a negligible PSII detachment during operation.

### 2.4 BPV Tests for Powering LEDs

**Figure 5:**
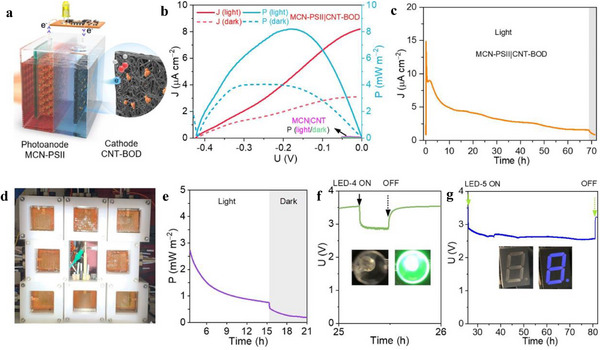
Large‐scale operation of the BPV device for electricity supply. a) Schematic illustration of the tandem BPV cell. An MCN‐PSII photoanode is wired to a CNT‐BOD cathode; both compartments are separated by a proton exchange membrane. b) J‐U curves recorded at 1 mV s^−1^ for MCN‐PSII (9 cm^2^)|CNT‐BOD (4 cm^2^). P was calculated as P = U × J. P of MCN (9 cm^2^)|CNT (4 cm^2^) is also calculated for comparison. c) Bias‐free photocurrent of MCN‐PSII (9 cm^2^)|CNT‐BOD (4 cm^2^) cell operating for over 69 h illumination, with the device connected to a 470 Ω resistor. d) Eight tandem MCN‐PSII (9 cm^2^)|CNT‐BOD (4 cm^2^) BPEC cells wired in series under light irradiation. e) Power output of the 8 BPV cells in the 3.5–21 h interval. Grey areas indicate dark periods. f,g) Tracing of U output from the 8 tandem BPV cells, with an LED light (f) and LED display (g). The photoanode half‐cell was N_2_ saturated and sealed, while the cathode half‐cell was exposed to air during the BPV test (AM 1.5 G, 100 mW cm^2^).

While electricity produced by photosynthetic microorganisms (mainly cyanobacteria, Table S3, Supporting Information) has been harvested for BPV applications,^[^ ^41^, ^42^ ^]^ they rely on complex intracellular electron transfer and metabolic regulation. This often results in slower, less predictable, and less controllable photocurrent responses. In contrast, PSII can directly drive water oxidation under light, without involving cellular metabolism, allowing for precise tuning of interfacial architecture and electron transfer, as demonstrated in this work. While PSII was rarely investigated for BPV applications, spinach‐extracted PSII also offers practical advantages in terms of availability, making it a preferred choice for applications compared to cyanobacteria. MCN‐PSII photoanodes enabled us to ultimately construct BPV devices for powering microelectronics (**Figure** **5**a). To this end, an MCN‐PSII photoanode was wired to a BOD well‐immobilized CNT (CNT‐BOD) film biocathode, whose XANES spectra (Figure S55, Supporting Information) confirm the presence of C and O functional groups, and SEM images (Figure S56, Supporting Information) show a well‐assembled CNT network with sufficient gaps for BOD encapsulation. Freeze treatment of the CNT‐BOD electrode (Figure S57, Supporting Information) further reduced the charge transfer resistance and enhanced ORR current. Both half‐reactions were separated by a proton exchange membrane due to different electrolyte solutions (see Experimental Section). CNT‐supported BOD has been previously established for oxygen reduction to water, as the cathodic half‐reaction in biofuel cells.^[^ ^43^ ^]^ As illustrated in Figure S56 (Supporting Information), the porosity of the CNT films suits the immobilization of BOD enzymes, whereas their conductivity makes them an excellent electrode material. While previous studies utilized CNT powders,^[^ ^43^ ^]^ here we introduced a flexible CNT film, which enabled a facile, standalone biocathode preparation.

As a reductase, BOD can perform the cathodic ORR effectively, unaffected by light irradiation.^[^ ^44^ ^]^ As shown in Figure S57 (Supporting Information), EIS spectra and cyclic voltammetry scans verified that freeze treatment induced a closer CNT‐BOD biointerface, yielding enhanced electrical communication and higher ORR current. The freeze‐treated CNT‐BOD electrode (4 cm^2^) delivered an initial limiting current of 224 µA under air, which gradually decayed to 170 µA after 20 scans at 5 mV s^−1^.

The power outputs of the assembled MCN‐PSII|CNT‐BOD BPEC cells were probed by current‐voltage (*J*‐*U*) curves at 1 mV s^−1^ scan rate (Experimental Section and Figure 5b; Figure S58, Supporting Information). Under irradiation, MCN‐PSII (9 cm^2^)|BOD‐CNT (4 cm^2^) and MCN‐PSII (33 cm^2^)|BOD‐CNT (10.5 cm^2^) of different sizes delivered comparable open circuit voltages (V_OC_, at 0 mA m^−2^) of 0.42 and 0.45 V, and power densities (P) of 7.8 and 8.2 mW m^−2^, respectively, whereas MCN|CNT cells (Figure 5b) displayed negligible V_OC_ and P values. We further adopted the MCN‐PSII (9 cm^2^)|BOD‐CNT (4 cm^2^) configuration for subsequent BPV tests.

Although the initial power output of BPV devices is often reported, their long‐term stability is equally important but remains largely underexplored in microorganism‐based systems^[^ ^45^ ^]^ (Table S3, Supporting Information). Here, we assessed the J output of this bias‐free BPV cell by connecting it to a 470 Ω resistor (Figure 5c), which yielded an initial power output of 9.3 mW m^−2^ under irradiation (Figure S59, Supporting Information). A gradual power decrease was observed beyond the first hour, maintaining only 0.1 mW m^−2^ after 69 h of continuous light irradiation. This behavior was due to the activity decay of both PSII and BOD electrodes, as indicated by Figure 4a,b and Figure S57b (Supporting Information). A power output was also observed in the dark, as commonly encountered in photosynthetic enzyme‐based BPV devices.^[^ ^46^ ^]^

BPV cells were next wired to various LED electronics to demonstrate the real‐world applicability of our systems (Figure S60 and Table S4, Supporting Information). Eight tandem MCN‐PSII (9 cm^2^)|BOD‐CNT(4 cm^2^) BPV cells were wired in series to generate enough voltage (Figure 5d), while a low current circuit was designed to generate LED light flashes (Experimental Section, Figures S61 and S62, Supporting Information). Following the earlier observed activity of MCN‐PSII photoanodes, the voltage output of tandem cells was also traced over 5 days, while performing subsequent BPV tests (Figure S63, Supporting Information). The serially connected BPV array produced a voltage of 3.45 V under irradiation (Figure S64a, Supporting Information), with an average value of 0.43 V for each cell, consistent with the measured 0.42 V for a single cell as shown in Figure 5b. As expected, voltage drops occurred when the BPV array powered the LED lights or LED display (Figure 5f,g; Figure S64, Supporting Information). Once disconnected, a fast voltage recovery was observed, demonstrating effective charge transfer. Light flashing (1 s^−1^) was clearly noticeable for LEDs 2, 4, 5, and 6, while a variable resistor (Figure S62, Supporting Information) was needed to draw more power from the BPV cells for LEDs 1 and 3.

The power output of the BPV array was evaluated by connecting it to a 4.7 kΩ resistor (Figure S64b,e,f, Supporting Information). The overall power output of the eight cells (8 × 9 cm^2^) decreased from 2.8 to 0.8 mW m^‒2^ in between the 3.5 and 15.3 h interval of irradiation (Figure 5e), which is comparable to the power output of 2.5–0.7 mW m^‒2^ observed in a single cell (9 cm^2^) over the same period (Figure S59, Supporting Information). The BPV array retained only 0.1 mW m^‒2^ after 132 h (Figure S65, Supporting Information). A lower power output was sustained for several hours after the light was turned off, which could also provide enough power to supply LED 5 (Figure S64d, Supporting Information). In terms of the maximum power output, this PSII‐based BPV device compares favorably to most previously reported microbial‐based BPVs (Table S3, Supporting Information). This indicates that similar PSII‐based BPV devices may soon supply a broader range of low‐power applications.

## 3 Conclusion

This proof‐of‐concept study demonstrates the potential of PSII photoanodes as versatile semi‐artificial platforms toward solar energy conversion. The introduction of protonated MCN as a porous scaffold allows PSII immobilization in vitro, enabling the assembly of MCN‐PSII Z‐scheme structure. Large‐area MCN‐PSII photoanodes are fabricated by introducing a spray‐freeze technique to establish a close biointerface with efficient charge transfer. Accordingly, 33 cm^2^ MCN‐PSII photoanodes demonstrate initial photocurrents in the mA range and an O_2_ Faradaic yield of 93.5 ± 8.5% over 5 h. A bias‐free BPEC device incorporating an MCN‐PSII photoanode and a CNT‐BOD cathode further sustains power output, showcasing the potential of this technology in powering small electronic devices. These findings not only advance the fundamental understanding of PSII‐based semi‐artificial systems but also establish the spray‐freeze method as a general and scalable platform for constructing large‐area electrodes with improved interfacial integration. However, long‐term operational stability remains a limiting factor, highlighting the need to improve robustness of PSII‐based devices.

## 4 Experimental Section

### Fabrication of Protonated MCN

MCN was synthesized by a hard‐templating method. According to the Stöber method,^[^ ^47^ ^]^ SiO_2_ nanospheres were first synthesized with the size of ≈300 nm via a sol‐gel process, using ammonia and TEOS in ethanol. SiO_2_ dispersed in ethanol was then vertically settled and self‐assembled into a hard template film. Dicyandiamide (0.60 g) was evenly spread onto 1.00 g SiO_2_ template film, and then annealed at 520 °C for 2 h, followed by annealing at 550 °C for 2 h under an N_2_ atmosphere. The resulting powder was etched by NH_4_HF_2_ (4 m) to remove the SiO_2_ template and obtain MCN. b‐CN was prepared from dicyandiamide by the same annealing procedure.

### Isolation of PSII Dimers from Spinach Leaves

PSII was isolated from the market‐sourced spinach according to the previously reported procedure.^[^ ^15^ ^]^ Each sub‐packaged PSII stock solution containing ≈1.1 mg Chl *a* mL^−1^ was stored at −80 °C. Typical absorption peaks of Chl *a* were observed at 434, 458, and 663 nm in the UV–vis diffuse reflectance spectrum of PSII dimer solution (Figure S1, Supporting Information), verifying its successful extraction from the thylakoid. The Chl *a* concentration (C_Chl_ *_a_*) could be obtained according to Equation (1).

(1) $$C_{\mathrm{Chl}\;a\;}\left(\mu g\;mL^{-1}\right)=8.02\;\times \;\left(A_{663}-A_{720}\right)+20.21\;\times \;\;\left(A_{645}-A_{720}\right)\;$$

where, A_645_, A_663_, and A_720_ refer to UV absorbance at 645, 663, and 720 nm, respectively.

### Fabrication of 0.196 cm^2^ MCN, b‐CN, MCN‐PSII, and b‐CN‐PSII Electrodes

To prepare MCN and b‐CN electrodes, 3 mg of MCN or b‐CN powder was suspended in a mixture of 250 µL isopropyl alcohol and 5 µL Nafion and then treated by ultrasonication for 30 min. After that, 5 µL of the suspension was drop‐casted onto a pre‐defined area (5 mm in diameter, 0.196 cm^2^) on FTO glass (1 × 1.5 cm^2^), which was repeated three times to form MCN or b‐CN film. A PSII electrode (5 mm in diameter, 0.196 cm^2^) was fabricated through the impregnation self‐assembly method. In detail, an MCN or b‐CN electrode was immersed vertically into PSII stock solution for the self‐assembly of PSII enzymes into the b‐CN or MCN pores. After 8 h, the electrode was gently lifted and dipped into PSII buffer solution (pH 6.5; containing 20 mm MES, 50 mm KCl, 3 mm CaCl_2_, and 5 mm MgCl_2_) to wash off PSII dimers that were loosely bound. The electrodes were then dried under air circulation for 4–6 h. All procedures were conducted in the dark at 4 °C and the prepared b‐CN‐PSII and MCN‐PSII electrodes were stored in a refrigerator at −80 °C before tests. This process may reinforce the binding between PSII and MCN or b‐CN.

### Preparation of Large‐Scale MCN‐PSII and MCN Electrodes

MCN powder of 40 mg was first suspended in a mixture of 10 mL isopropyl alcohol and 200 µL Nafion and ultrasonicated for 30 min. The catalyst ink was sprayed using an airbrush (Figure S3a, Supporting Information) onto a 6 × 6 cm^2^ FTO glass with a predefined area of 6 × 5.5 cm^2^ to obtain an MCN electrode (Figure 1b). A 33 cm^2^ MCN‐PSII electrode was next prepared by spraying 2 mL PSII stock solution onto the 6 × 5.5 cm^2^ MCN electrode, followed by incubation at 4 °C under dark for 16 h. 3 × 3 (9) cm^2^ MCN and MCN‐PSII electrodes were prepared in a similar way by loading the same ratios of MCN and PSII onto 3 × 3.5 cm^2^ FTO glass. MCN‐PSII electrodes were then stored at –80 °C for freeze treatment. Prior to PEC studies, an MCN‐PSII electrode was defrosted at 4 °C in dark for 10 min, and the electrode was gently rinsed by PSII buffer solution to get rid of loosely bound species (Figure S66, Supporting Information).

### Preparation of CNT‐BOD Cathode

The as‐received CNT film was first treated with acetone and water. Before BOD immobilization, the CNT film was modified by 1‐pyrenecarboxylic acid (PC), which could act as a promoter layer to enhance the oxygen reduction activity of BOD on the electrode surface.^[^ ^43^ ^]^ 2 × 2 (4) cm^2^ or 3.5 × 3 (10.5) cm^2^ CNT films were modified with 50 or 100 µL of 2.5 mm PC (dissolved in ethanol) and dried for ≈2 h. The PC‐modified CNT film was then rinsed with ethanol to remove unbound π‐systems and dried under an N_2_ stream. For the immobilization of BOD, 50 µL BOD stock solution (0.625 unit in 0.1 m citrate phosphate buffer, pH 7) was spread evenly onto the surface of the PC‐modified CNT film, allowing it to incubate for 12 h at 4 °C and the obtained CNT‐BOD films (4 or 10.5 cm^2^) were stored at −80 °C before tests.

### PEC Photoanode Measurements Under Air

To obtain the PEC profiles of MCN, b‐CN, MCN‐PSII, and b‐CN‐PSII photoanodes, PEC tests were carried out under air at room temperature using a Zennium workstation (Zahner, Germany), in a three‐electrode configuration with a Pt spiral wire as the counter electrode and KCl‐saturated Hg/Hg_2_Cl_2_ electrode (SCE) as the reference electrode. A PSII buffer solution (pH 6.5, composition given above) was employed as the electrolyte for DET tests. For MET‐related tests, 1 mm DCBQ was added to the PSII buffer solution. A total reflection mirror was used to convert the vertical light source from an AM 1.5G simulator (TriSOL, OAI) to horizontal illumination. The light intensity reaching the PEC cell was measured as 80 mW cm^−^^2^ by a power meter. Under solar light irradiation with 20 s dark/light intervals, DET and MET photocurrents were recorded at initially detected open circuit potential values of the photoanodes, 0.40 and 0.60 V versus SCE. All photoanodes were back‐irradiated. Net photocurrent densities, J_DET, net_ and J_MET, net_, were defined as the second photocurrent peak, which was baseline‐corrected by subtracting the background dark current.^[^ ^8^ ^]^ MET EIS spectra were obtained under dark or light (80 mW cm^−2^) illumination from 100 kHz to 100 mHz in the 1 mm DCBQ added PSII buffer solution.

### Photocurrent Action Spectra Under Single‐Wavelength Illumination

DET and MET photocurrents of 0.196 cm^2^ electrodes were measured in PSII buffer solutions with or without 1 mm DCBQ, under air, at different applied potentials. Light was provided by a CEL‐SLF300 monochromator (Ceaulight, China). The wavelength‐dependent J_DET, net,_ and J_MET, net_ spectra were obtained after subtracting the corresponding dark currents. IPCE were calculated using Equation 2, where *I*_e_ – electron flux at the external circuit (mol m^−2^ s^−1^), *I*_λ_ – incident photon flux (mol m^−2^ s^−1^), *h* – Plank constant(6.626 × 10^−34^ J s), *c* – speed of light (3.00 × 10^8^ m s^−1^), *J*_net_ – net photocurrent density (A m^−2^), F‐ Faraday constant (96 485.3321 s A mol^−1^) e – electron charge (1.602 × 10^−19^ C), N_A_– Avogadro constant (6.022 × 1023 mol^−1^), *λ* – irradiation wavelength (m) and *E*_e_ – light intensity flux (irradiance) (W m^−2^).

(2) $$\mathrm{IPCE}=\frac{I_{e}}{I_{\lambda }}=\frac{\frac{J_{net}}{F}}{\frac{\lambda E_{e}}{N_{A}hc}}=\frac{hc\;\times \;J_{net}}{e\lambda E_{e}}$$

### PSII Loading Determination

To wash off the electrode film from the FTO substrate, b‐CN‐PSII or MCN‐PSII electrodes were immersed in 3.5, 50, or 100 mL acetone/water (4:1) solutions, depending on the electrode size of 0.196, 9, or 33 cm^2^, respectively. The obtained suspension was then centrifuged at 8000 rpm for 5 min and the UV spectrum of the supernatant was measured. Chl *a* concentration in the solution was then calculated according to Equation 1. The loading amount of PSII could also be obtained since a PSII dimer had 35 Chl *a* molecule (molar mass: 893.51 g mol^−1^).

### PEC Tests of MCN‐PSII and MCN Photoanodes Under N_2_ Atmosphere

PEC tests were conducted in PSII buffer solution with 1 mm DCBQ, which was purged with N_2_ for 30 min to remove O_2_ dissolved in the solution and from the headspace of the reactor (Figure 1d; Figure S3b, Supporting Information). Long‐term tests were performed in a three‐electrode configuration with a 33 cm^2^ MCN‐PSII photoanode, SCE reference electrode, and Pt plate (4 × 4 cm^2^) as a counter electrode. The Pt electrode was separated from MCN‐PSII and SCE by a Nafion 117 perfluorinated membrane to avoid product or impurity crossover. The PEC test was performed at 0.60 V versus SCE in a sealed reactor, under simulated solar irradiation (AM 1.5G, 80 mW cm^−2^, 12 h light/6 h dark cycles). O_2_ quantification was performed under N_2_ flow using a mass flowmeter (Sevenstar Mass flowmeter D07‐7) and gas chromatograph (GC, Agilent 8890). Inlet 1 or 2 of the electrolytic cell was connected to the mass flowmeter, while outlet 7 was connected to the GC (Figure S3b, Supporting Information). The mass flowmeter supplied N_2_ carrier gas at a 1.2 sccm (1.2 mL min^‒1^) flow rate. The carrier gas transported products from the headspace of the PEC reactor to the column of the GC, which sampled the gas mixture every 13 min. The TOF was calculated according to Equation (3), where M(O_2_) are the moles of O_2,_ M(PSII monomer) are the moles of PSII monomers loaded on an electrode, and t is the time.

(3) $$\mathrm{TOF}=\frac{M\left(O_{2}\right)}{M\left(PSII\;monomer\right)*t}$$

### Time‐Resolved Photoluminescence (TRPL) Fitting and Lifetime Calculation

TRPL decay curves were analyzed using a biexponential decay model:^[^ ^48^ ^]^

(4) $$I\left(t\right)=A_{1}e^{-t/\tau_{1}}+A_{2}e^{-t/\tau_{2}}$$

The average lifetime (τ_avg_) was calculated based on normalized amplitude values using:

(5) $$\tau_{avg}=\frac{A_{1}\tau_{1}+A_{2}\tau_{2}}{A_{1}+A_{2}}$$

### Wiring MCN‐PSII Photoanodes with CNT‐BOD Cathodes for BPV Tests

CNT‐BOD electrodes were characterized by cyclic voltammetry tests in air from −0.10 to 0.70 V versus SCE, at a scan rate of 10 mV s^−1,^ in a three‐electrode system with an SCE and Pt plate, in PSII buffer solution. An MCN‐PSII photoanode was wired to a CNT‐BOD cathode in a two‐compartment BPEC cell, separated by a Nafion 117 perfluorinated membrane. N_2_ saturated PSII buffer solution (pH 6.5) with 1 mm DCBQ was used as the electrolyte in the sealed MCN‐PSII photoanode compartment to retain PSII photoactivity,^[^ ^15^ ^]^ while PSII buffer solution was exposed to air in the cathodic compartment to sustain the oxygen reduction reaction. The BPV performance of the BPEC cells, i.e., MCN‐PSII (9 cm^2^)|CNT‐BOD (4 cm^2^), MCN (9 cm^2^)|CNT (4 cm^2^), or MCN‐PSII (33 cm^2^)|BOD‐CNT (10.5 cm^2^), were first characterized by applying a linear voltage sweep from V_OC_ to 0 V at 1.0 mV s^−1^, under simulated solar light illumination (AM 1.5G, 80 mW cm^−2^) or under dark. The delivered P was derived from the following relationship in Equation 6.^[^ ^44^ ^]^

(6) P=J×U

To measure the long‐term, bias‐free BPV performance, an MCN‐PSII (9 cm^2^)|CNT‐BOD (4 cm^2^) BPEC cell was connected to a 470 Ω external resistor. The BPV voltage output was traced on a Zennium workstation for 69 h, under simulated solar light irradiation (AM 1.5G, 80 mW cm^−2^).

### Serially Connected Tandem BPEC Cells for Powering LED Electronics

A low current LED flasher circuit (Figure S62, Supporting Information) was fabricated by the Lab and Workshop team from the Faculty of Sciences, Engineering and Technology, The University of Adelaide by following the blueprint available on DiscoverCircuits.com, designed by David Johnson. This circuit draws power from a 3 V supply with small current drain, allowing for the generation of flashlights. Eight BPEC cells were connected in series; the reactors were stacked up (Figure 5d), with all reactor windows confined in a 20 × 20 cm^2^ area. A fly‐eye lens was added to a 300 W Xe lamp with a horizontal light source to obtain an irradiation area of 20 × 20 cm^2^ and a light intensity of AM1.5G, 100 mW cm^−2^. The voltage of the BPV array was traced over 5 days under illumination. LED lights (⌀ 3–10 mm) or LED displays were connected to the designed electronic circuit and BPV array at different times for flashing tests. The BPV array was connected to an external resistor (4.7 kΩ) to record the power output at various times.

## Conflict of Interest

The authors declare no conflict of interest.

## Author Contributions

H.Z. and W.T. contributed equally to this work. H.Z. and S.W. conceived and designed the project. H.Z. and W.T. prepared the samples and designed the electrolytic cell and circuit. H.Z. conducted photoelectrocatalytic tests and microscopy characterizations. J.L. conducted some optical characterizations. P.Z. performed XPS; G.S. provided supervision. H.Z. and W.T. wrote the manuscript with input from S.R., H.S., E.C., V.A., and S.W. All the authors discussed the results and commented on the manuscript. S.W. supervised the project.

## Supporting information



Supporting Information

## Data Availability

The data that support the findings of this study are available from the corresponding author upon reasonable request.
