# Supplementary material for: Photosystem II‐Carbon Nitride Photoanodes for Scalable Biophotoelectrochemistry
Source: Adv Mater. 2025 Aug 15;38(3):e08813. doi: 10.1002/adma.202508813 (PMC12801365; doi:10.1002/adma.202508813)
Supplement: Supplementary file 1 — Supporting Information [file ADMA-38-e08813-s001.docx]

Supporting Information

Photosystem II-Carbon Nitride Photoanodes for Scalable Biophotoelectrochemistry

Huayang Zhang, Wenjie Tian, Jingkai Lin, Peng Zhang, Guosheng Shao, Sai Kishore Ravi, Hongqi Sun,^*^ Emiliano Cortés,^*^ Virgil Andrei,^*^ and Shaobin Wang^*^

H. Zhang, W. Tian, J. Lin, S. Wang

School of Chemical Engineering, The University of Adelaide, Adelaide, Australia
E-mail: [shaobin.wang@adelaide.edu.au](mailto:shaobin.wang@adelaide.edu.au)

W. Tian, E. Cortés

Nanoinstitute Munich, Faculty of Physics, Ludwig-Maximilians-Universität München, Munich, Germany

E-mail: [emiliano.cortes@lmu.de](mailto:emiliano.cortes@lmu.de)

P. Zhang, G. Shao
School of Materials Science and Engineering, Zhengzhou University, China

S.K. Ravi

School of Energy and Environment, City University of Hong Kong, Kowloon, Hong Kong

H. Sun

School of Molecular Sciences, The University of Western Australia, Perth, Australia

E-mail: [hongqi.sun@uwa.edu.au](mailto:hongqi.sun@uwa.edu.au)

V. Andrei

School of Materials Science and Engineering, Nanyang Technological University, Singapore

Yusuf Hamied Department of Chemistry, University of Cambridge, Cambridge, UK

E-mail: virgil.andrei@ntu.edu.sg

**Experimental section**

**Chemicals.** Most chemicals, 2,6-dichloro-1,4-benzoquinone (DCBQ, 98.0%), methanol (≥99.9%), acetone (≥99.9%), 2-(N-morpholino)ethanesulfonic acid (MES, ≥99%), 3-(3,4-dichlorophenyl)-1,1-dimethylurea (DCMU, ≥98%), dicyandiamide (>99.0%), calcium chloride (CaCl_2_ ≥99.9%), magnesium chloride (MgCl_2_≥99.9%), potassium hydroxide (KOH ≥90.0%), ammonium hydrogen difluoride (NH_4_HF_2_ ≥99.9%), citrate phosphate buffer tablets, 1-pyrenecarboxylic acid, Nafion™ 117 perfluorinated membrane, tetraethyl orthosilicate (TEOS, ≥90.0%) and FTO-coated glass slides (8 Ω sq^−1^) were purchased from Sigma-Aldrich and used without further purification unless otherwise noted. Potassium chloride (KCl ≥90.0%) was purchased from Alfa Aesar. CNT film was received from XFNANO (Nanjing, China). Bilirubin oxidase (BOD) from *Myrothecium verrucaria* was directly purchased from Sigma-Aldrich and suspended in the citrate-phosphate buffer (0.1 M, pH 7.2) solution. LED lights and displays were purchased from Jaycar Electronics, Australia.

**Material characterization.** SEM experiments were performed with FEI QUANTA 450 FEG environmental SEM at acceleration voltages of 10-30 kV. HAADF-STEM images were acquired on FEI Titan Themis 80-200 at 200 kV. X-ray absorption near-edge structure (XANES) spectra were obtained using the Soft X-ray Spectroscopy beamline at the Australian Synchrotron. XPS was performed on a Kratos Axis Ultra X-ray Photoelectron Spectrometer. The in-situ irradiation XPS was measured via a Shimadzu AXIS Supra instrument with Al Kα irradiation. CLSM images were taken on an Olympus FV3000 Confocal Microscope. UV-vis diffuse reflectance spectra were measured on a Cary 100 UV-vis spectrophotometer (Agilent, US). XRD patterns were obtained on a Rigaku X-ray Diffractometer. Time-resolved photoluminescence (TRPL) spectra were recorded at room temperature using an FLS1000 spectrometer (excitation of 350 nm, emission of 470 nm, Edinburgh Instrument). The steady-state photoluminescence (PL) spectra were obtained using an RF-5301PC spectrofluorophotometer (Shimadzu). Zeta potential was obtained using Zetasizer Nano Series (Malvern). Solid-state NMR spectroscopy was recorded using a Bruker Avance Neo 400WB Solid-State NMR Spectrometer (Bruker).

**Figure S1** UV-vis absorbance spectrum of PSII dimer stock solution.


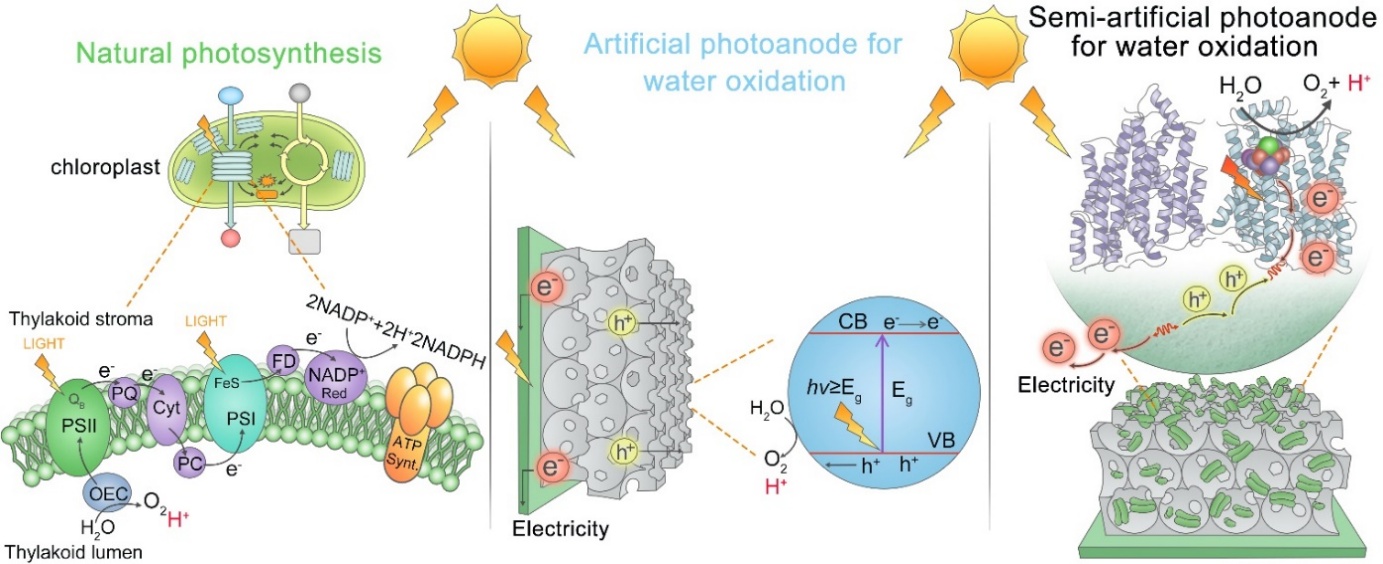


**a**

**b**

**c**

**Figure S2** Schematic illustration of natural, artificial, and semi-artificial processes. (a) Biological photosynthesis for water oxidation and chemical synthesis: oxygenated photosynthesis has evolved over billions of years to form mature enzymatic catalytic mechanisms.^[1]^ Many enzymes can catalyze chemical reactions with high turnover frequency (TOF), high selectivity, and minimal energy loss. (b) Artificial photosynthesis on a semiconductor electrode for PEC water oxidation and solar-to-electricity conversion. (c) Semi-artificial photosynthesis that employs PSII photoanode for PEC water oxidation and electricity generation.

**a**


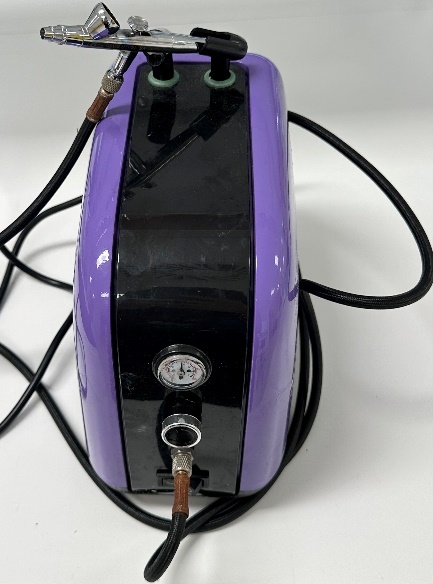

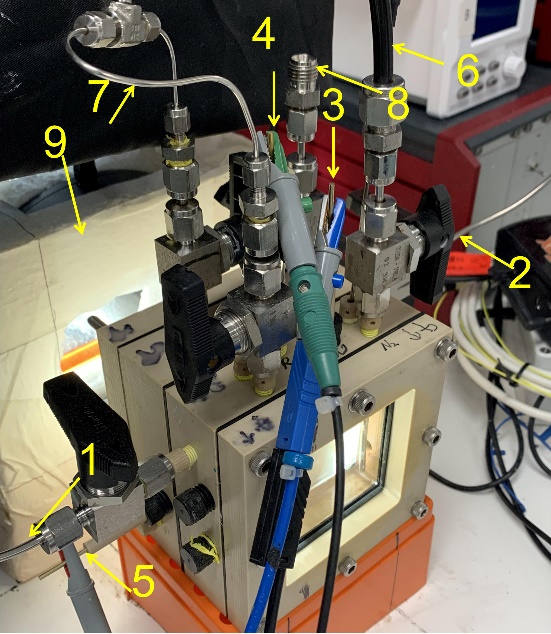


**b**

**Figure S3** The setup used for large-scale PEC tests. (a) Iwata 2spray air compressor with airbrush for spray coating of large-scale electrodes. (b) A customized three-electrode cell for PEC tests of 6×5.5 cm^2^ MCN-PSII photoanodes under horizontal illumination. 1,2: inlet of mass flowmeter; 3: working electrode; 4: SCE reference electrode; 5: Pt plate counter electrode; 6: N_2_ purging inlet; 7: outlet that can be connected to a GC system; 8: N_2_ purging outlet; 9: customized large-caliber optical-electronic shutter (shutter diameter: 100 mm), which can be switched on and off automatically.


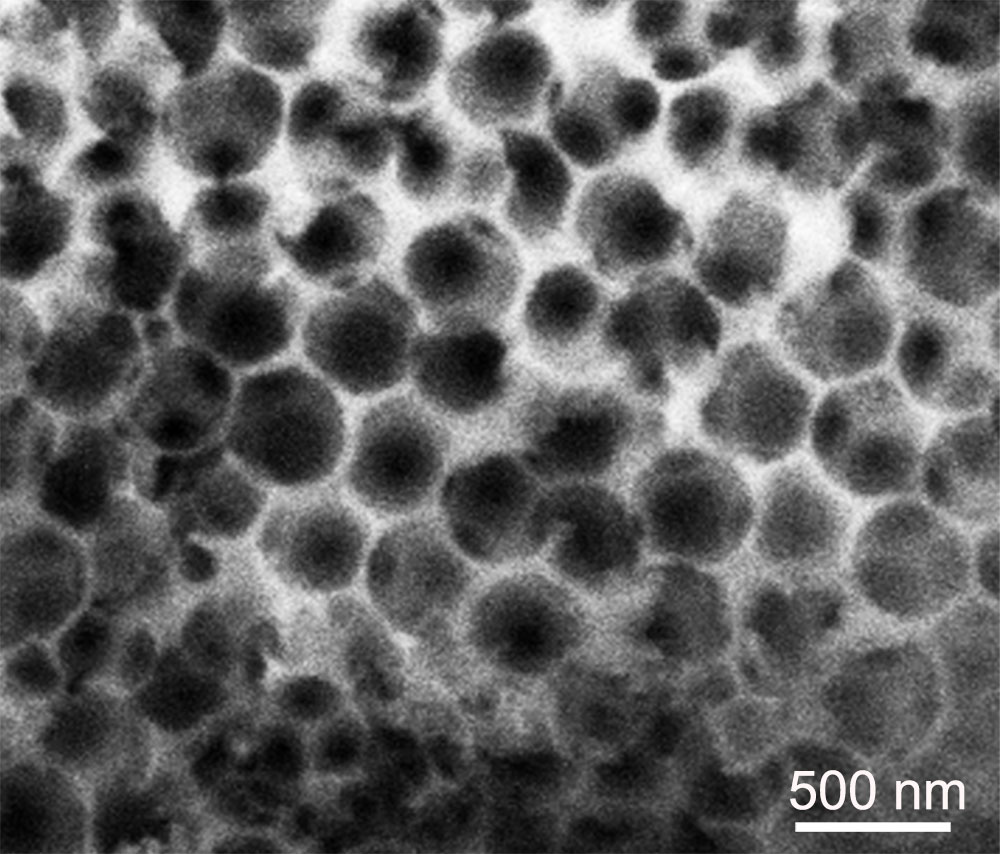


**Figure S4** Magnified SEM image of MCN acquired at 15 kV accelerating voltage condition.


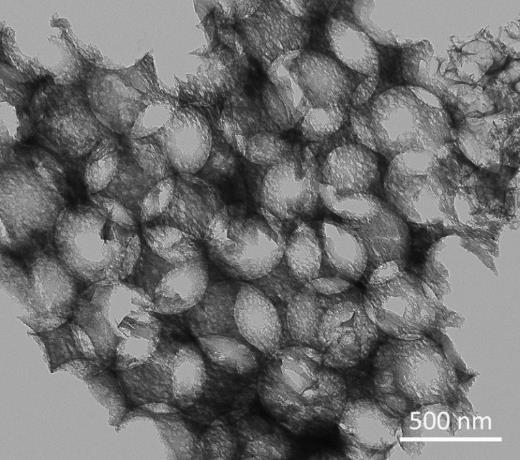

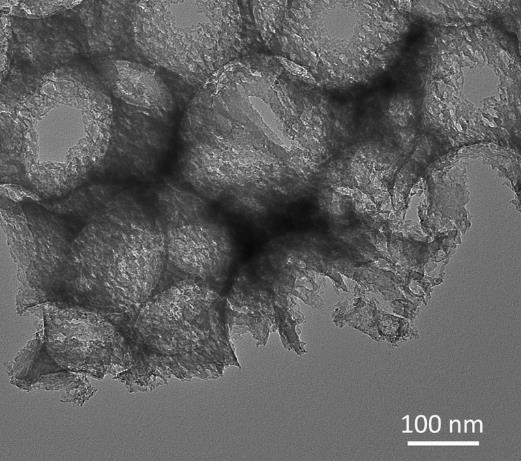


**b**

**a**

**Figure S5** TEM images of MCN.


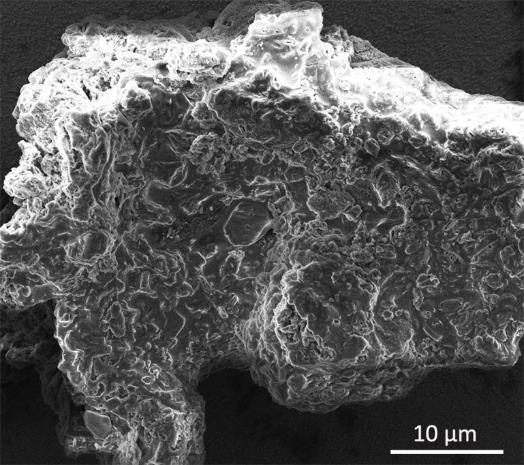


**Figure S6** SEM image of b-CN.


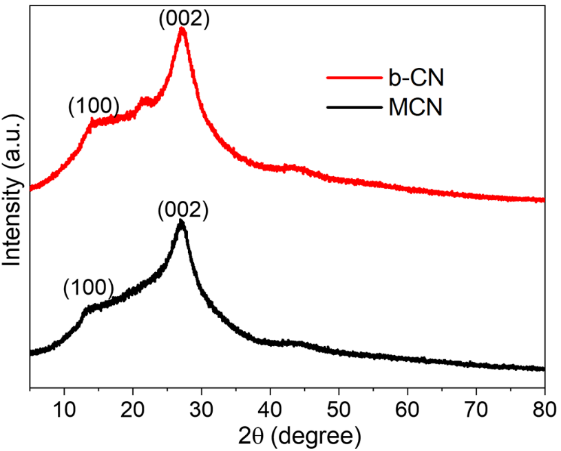


**Figure S7** Powder XRD patterns of b-CN and MCN. X-ray powder diffraction (XRD) patterns of MCN and b-CN reveal two characteristic (100) and (002) diffraction peaks, which are associated with the in-plane structural packing motif and the interplanar stacking of conjugated aromatic rings of carbon nitride, respectively^[2]^.

**Figure S8** FT-IR spectra of b-CN and MCN powder. FT-IR spectra of b-CN and MCN show similar functional groups of triazine, CN cycle and ‒NH/‒NH_2_ groups.^[3,4]^


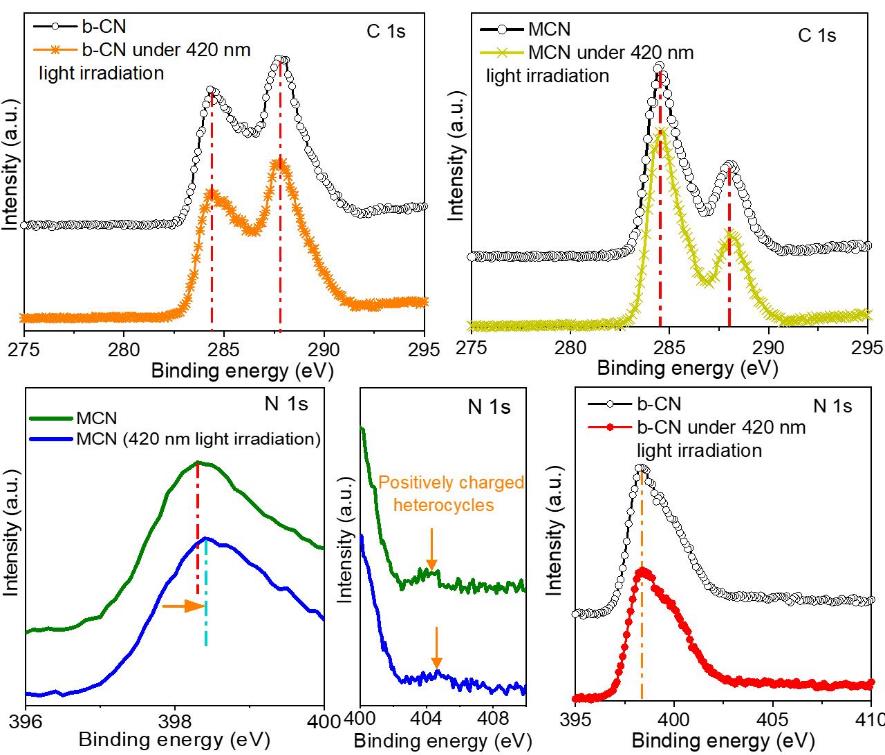


**a**

**b**

**c**

**d**

**e**

**Figure S9** High-resolution XPS spectra. (a, b) C 1s with and without in situ 420 nm light irradiation. (c-e) N 1s with and without in situ 420 nm light irradiation.

**a**

**b**

**Figure S10** Protonation of b-CN and MCN. (a) ^1^H solid-state NMR spectra for b-CN and MCN. (b) Zeta potentials of b-CN, MCN and PSII. ^1^H NMR spectra show the two major peaks at ~9.0 ppm (amino groups) and 4.0 ppm (residual water) for b-CN.^[3]^ The peak position of 4.0 ppm shifts to 4.6 ppm for MCN, which indicates that it becomes highly hydrophilic with the formation of hydrogen bonding with the adsorbed water molecules. The amino group peak of MCN shows a low-field shift compared to b-CN, suggesting the formation of hydrogen bond. The intensity of amino group peak decreased, suggesting enhanced proton exchange process^[4,5]^ and the protonation. Other additional peaks at 7.0 and 1.0 ppm in MCN are assigned to protons in ‒NH‒ and ‒NH_2_.^[6]^ Zeta potential of b-CN is about ‒0.54 mV, and MCN is about 0.42 mV, further confirming the protonation of MCN, which is beneficial for anchoring negatively charged PSII (‒7.60 mV). For comparison, we applied the same 4M NH_4_HF_2_ etching treatment on b-CN. The resulting zeta potential of b-CN changed to –0.28 mV, which did not result in a zeta potential as positive as that of MCN (0.42 mV). To further investigate, we treated MCN with 1 M NaOH, resulting in a Zeta potential change to –0.65 mV. While OHˉ adsorption may affect the Zeta potential, the observed shift is consistent with the known protonation/deprotonation behavior of g‑C_3_N_4_ materials. For example, mesoporous g‑C_3_N_4_ exhibits an apparent p*Kₐ* = 6.60 ± 0.3,^[5]^ above which deprotonation of surface –NH/–NH_2_ groups dominates, leading to a net negative surface charge. Given that the NaOH treatment was at pH ≫ p*K*_a_, the negative zeta potential is likely a result of surface deprotonation.

**b**

**a**

**Figure S11** Photoluminescence (PL) spectra. (a) Transient PL spectra. (b) Steady-state PL spectra. The spectra indicate improved charge transfer and separation within the MCN.

**Figure S12** Biexponential fitting results for time-resolved photoluminescence (TRPL) decay curves of (a) b-CN and MCN, (b) b-CN-PSII with and without freeze treatment, and (c) MCN-PSII with and without freeze treatment. All decay curves were fitted using a biexponential model. Fitting parameters are shown in the insets. Compared to bulk b-CN, MCN exhibits a shorter τ₁ and a slightly reduced τ_avg_, indicating more efficient charge transfer pathways. Upon PSII immobilization, τ_avg_ is further reduced, especially after freeze-treatment, suggesting enhanced interfacial electron transfer due to improved PSII integration and stronger electronic coupling.

(a)

(b)

(c)

2. 85

2. 85

2. 85

2. 85


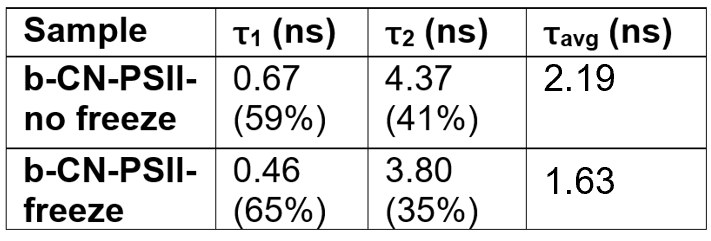

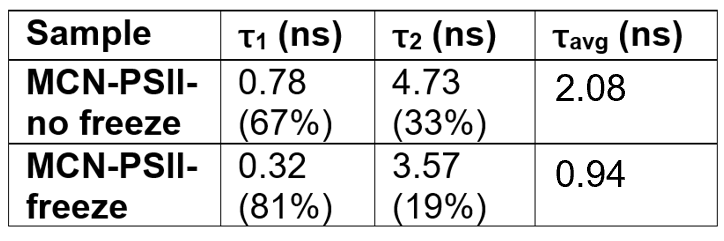

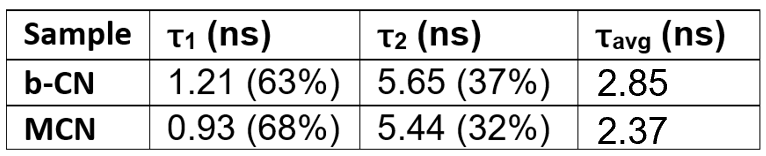

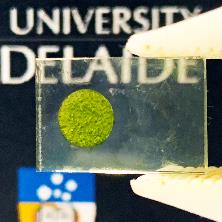

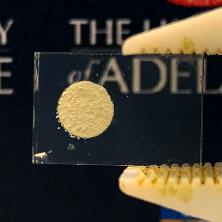


**a**

**b**


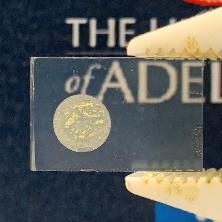

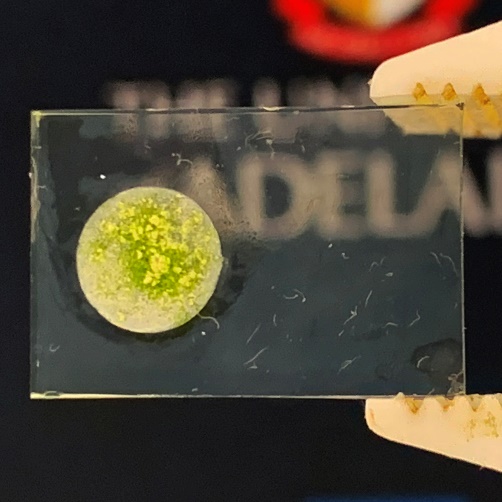


**c**

**d**

**Figure S13** Photographs of 0.196 cm^2^ electrodes. (a) MCN. (b) MCN-PSII. (c) b-CN. (d) b-CN-PSII. Images reveal the improved film-forming and PSII-anchoring capability of MCN over b-CN. (*The University of Adelaide name and logo are reproduced with permission from the University of Adelaide.*)


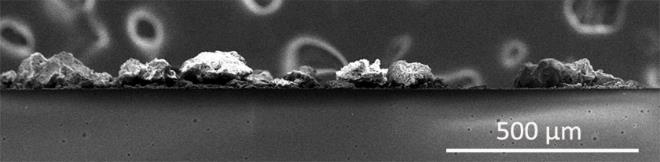

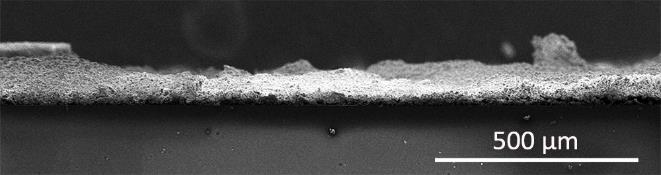

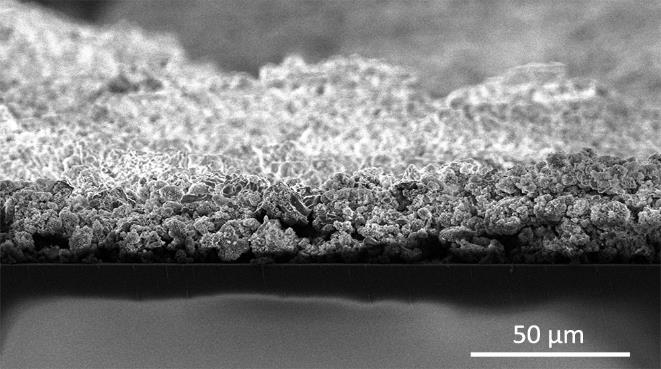


**a**

**b**

**c**

**Figure S14** Cross-sectional views of electrodes. (a) b-CN. (b, c) MCN.


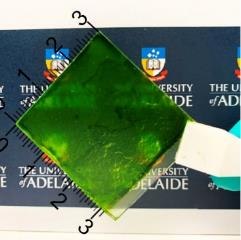

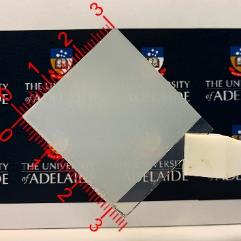

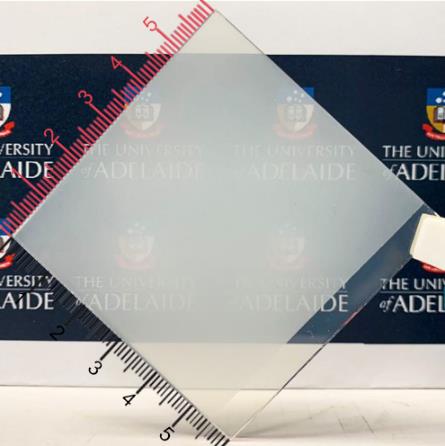


**a**

**b**

**c**

**Figure S15** Electrode photographs. (a) MCN. (b) MCN-PSII electrode of 9 cm^2^. (c) MCN electrode of 33 cm^2^ prepared by a spray coating approach. (*The University of Adelaide name and logo are reproduced with permission from the University of Adelaide.*)


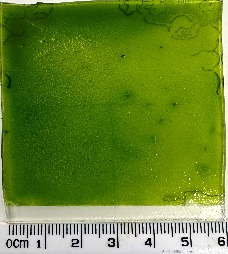

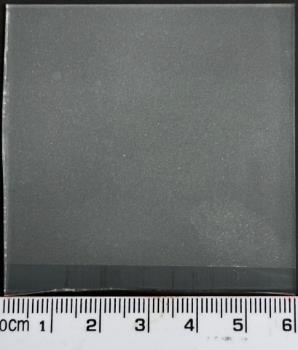


**a**

**b**

**Figure S16** Photographs of 33 cm^2^ electrodes prepared by a spray coating approach. (a) b-CN. (b) b-CN-PSII.


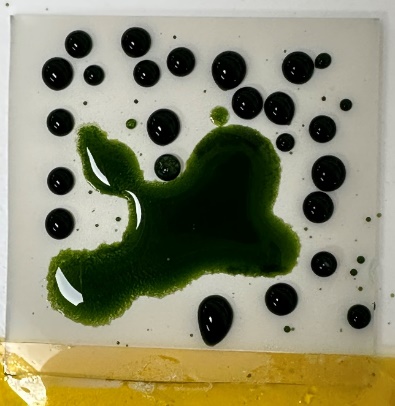


**Figure S17** Drop-casting of PSII solution onto a 33 cm^2^ MCN electrode.


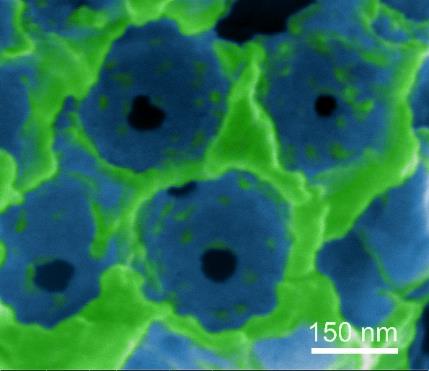


**Figure S18** Representative colorized SEM image of 0.196 cm^2^ MCN MCN-PSII electrode. A platinum layer (about 10 nm in thickness) was deposited on MCN-PSII to obtain a high-quality image.


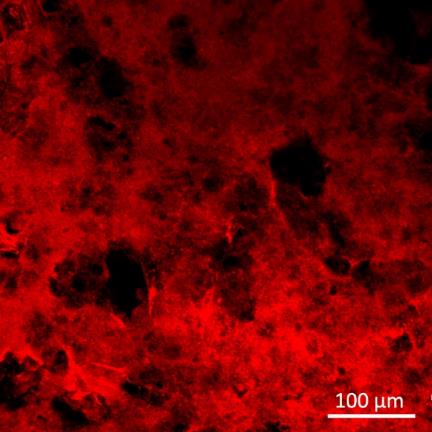


**Figure S19** CLSM image of 0.196 cm^2^ MCN-PSII electrode, with excitation at 559 nm. Dark patches correspond to areas where MCN clusters have not been deposited (see Figure S14b, c).


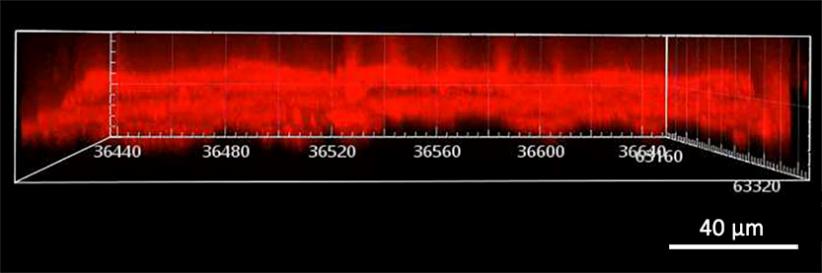

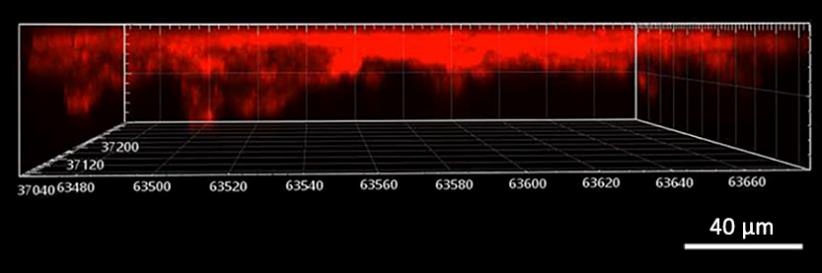


**b**

**a**

**Figure S20** CLSM images of the 33 cm^2^ electrode surface. (a) MCN-PSII electrode. (b) b-CN-PSII electrode.


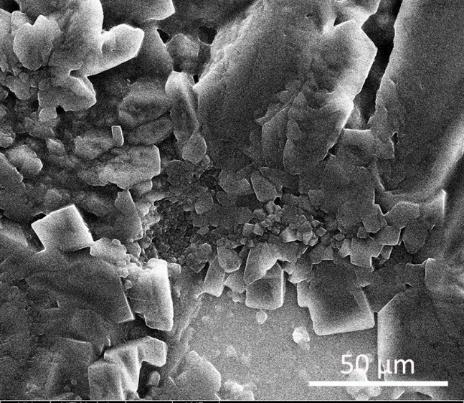

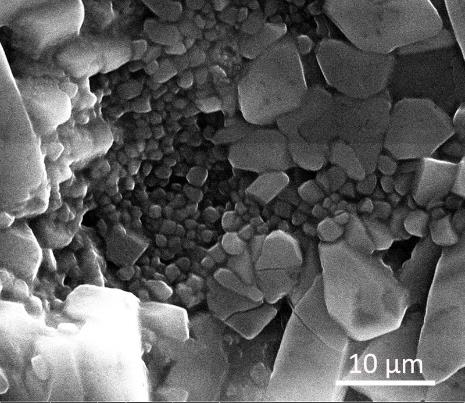


**b**

**a**

**Figure S21** SEM images of the 33 cm^2^ b-CN-PSII electrode surface.


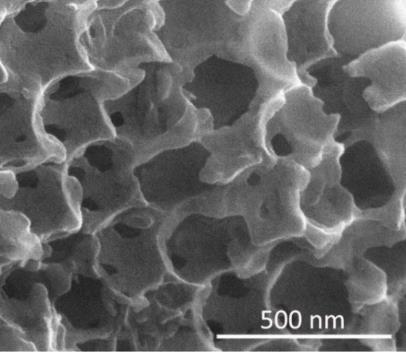

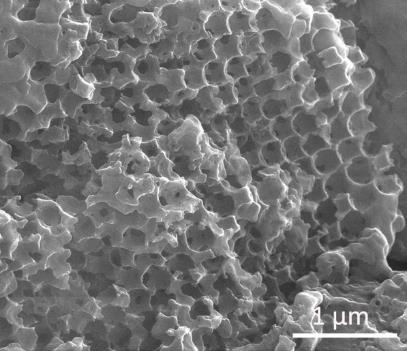

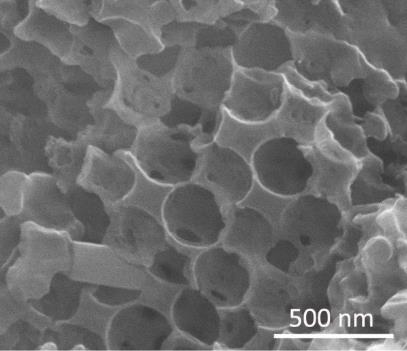

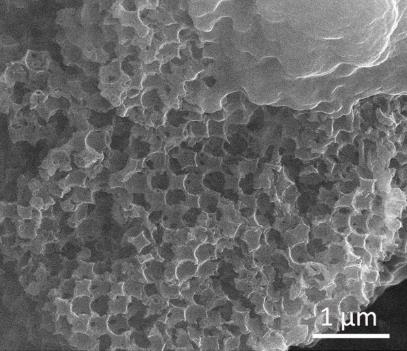


**c**

**b**

**a**

**d**

**Figure S22** SEM images of the 33 cm^2^ electrode surface. (a, b) MCN-PSII, no freeze treatment. (c, d) MCN-PSII, freeze treatment.


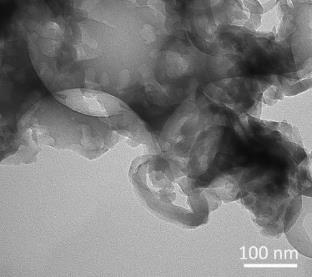

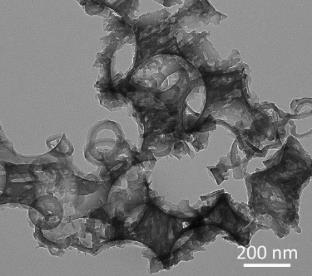

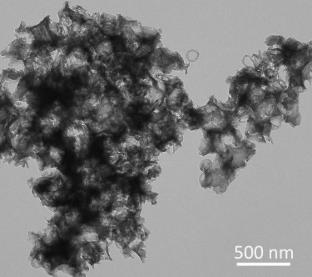

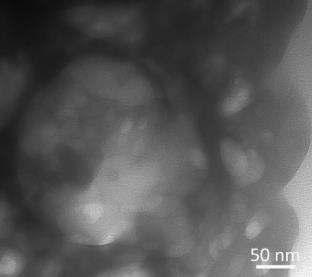

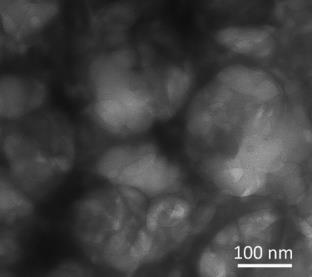

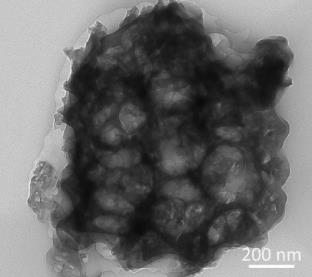


**c**

**b**

**a**

**f**

**e**

**d**

PSII

PSII

PSII

PSII

PSII

PSII

**Figure S23** TEM images. (a-c) MCN-PSII, no freeze treatment. (d-f) MCN-PSII, freeze treatment.

**c**

**b**

**a**

**e**

**d**

**Figure S24** Electrochemical characterization of 9 cm^2^ MCN-PSII and MCN electrodes. (a) LSV curves of MCN-PSII with no freeze treatment were recorded at a scan rate of 10 mV s^‒1^. (b) LSV curves of MCN-PSII with freeze treatment at a scan rate of 10 mV s^‒1^. (c) Tafel plots under light irradiation. MCN-PSII (9 cm^2^) electrodes were cut from the 33 cm^2^ electrode, for freeze treatment and no freeze treatment tests. MCN-PSII exhibits better LSV stability for 5 consecutive scans after freeze treatment. (d) MET EIS spectra of MCN with and without freeze treatment under light and no irradiation. (e) LSV curves of MCN with or without freeze treatment at a scan rate of 10 mV s^‒1^.

**c**

**b**

**a**

**d**

**Figure S25** Electrochemical characterization for 9 cm^2^ b-CN-PSII electrodes. (a) LSV curves recorded at a scan rate of 10 mV s^‒1^. (b) Calculated Tafel plots in the light. b-CN-PSII (9 cm^2^) electrodes were cut from the 33 cm^2^ electrode, for freeze treatment and no freeze treatment tests. (c) MET EIS spectra in the light and dark. (d) Fitted EIS spectra, based on the equivalent circuit shown in Figure S26.


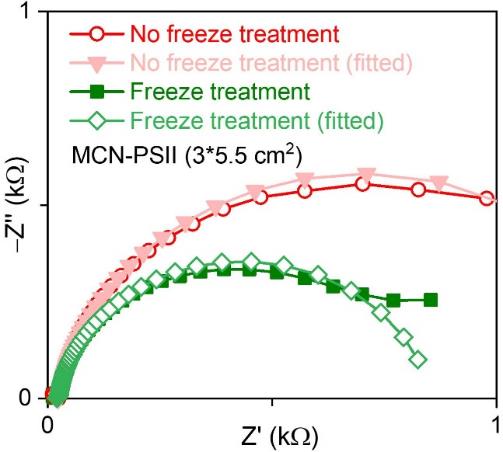


**Figure S26** EIS spectra of MCN-PSII electrodes (3×5.5 cm^2^) under irradiation. One 33 (6×5.5) cm^2^ MCN-PSII photoanode was cut into two half-electrodes (3×5.5 cm^2^). One half-electrode was freeze treated, while the other was not. The two half-electrodes were used for the MET EIS tests. The spectra were fitted based on Figure S27.


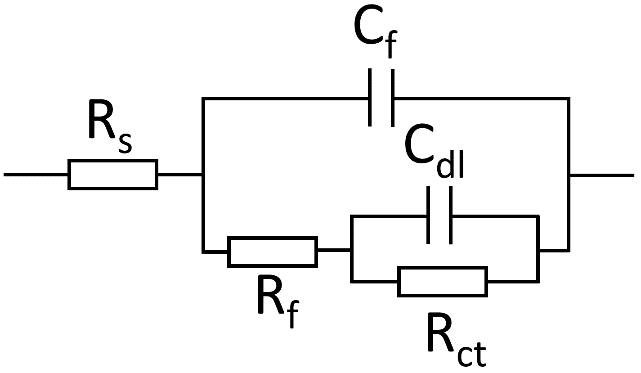


**Figure S27** Equivalent circuit for fitting EIS spectra. R_s_ - solution resistance, R_f_ - film resistance or surface layer resistance, R_ct_ - charge transfer resistance, C_dl_ - double layer capacitance or interfacial capacitance, C_f_ - film capacitance or surface layer capacitance.


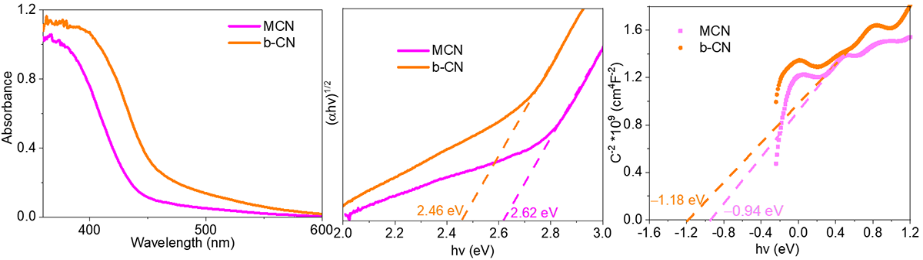


**a**

**b**

**c**

**Figure S28** Optical properties of MCN and b-CN powders. (a) UV-vis diffuse reflectance spectra. The light absorption of MCN powder slightly decreases in the visible light region compared with b-CN, possibly due to the lighter color. (b) Transformed Kubelka-Munk functions versus photon energy, based on (a). The bandgap energy values of b-CN and MCN are calculated to be 2.46 and 2.62 eV, respectively. (c) Mott-Schottky plots of b-CN and MCN. The flat band potential (E_fb_) values of b-CN and MCN are estimated to be −1.18 and −0.94 V, respectively, versus normal hydrogen electrode (NHE, pH=6.5). The values can be considered as the bottom of the conduction band (CB) for n-type semiconductors,^[7]^ i.e., b-CN or MCN in this work. Hence the corresponding valence band (VB) positions of b-CN and MCN were calculated as 1.28 and 1.68 V, respectively.


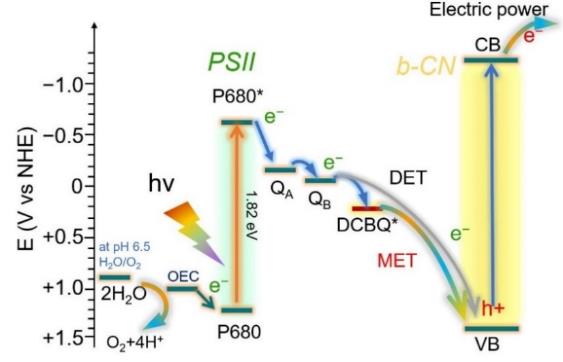


**Figure S29** Energy level diagram. Energy level diagram of b-CN-PSII electrode for the cascade DET or MET processes under irradiation. Like the energy level diagram of MCN-PSII (Figure 3a), the electrons on Q_B_ donor sites of PSII own a large driving force to inject into the VB of b-CN, forming b-CN-PSII Z-scheme structure, leading to improved charge separation in b-CN. As illustrated in Figures 3a and S28, under illumination, an electron in PSII is excited from P680 chromophore to pheophytin and plastoquinone A (Q_A_), generating the excited P680^+^ state (+1.19 V vs. NHE). P680^+^ then oxidizes tyrosine to extract electrons (or protons) in four photochemical cycles from the Mn_4_O_x_Ca oxygen-evolving complex (OEC) that binds water, catalyzing the oxidation of adsorbed water molecules for O_2_ evolution. Meanwhile, the electrons move to Q_A_ (−0.14 V vs. NHE) and subsequently to the plastoquinone B (Q_B_, −0.06 V vs. NHE) terminal site on the stromal side of the PSII.^[8,9]^ In contrast, electrons are captured in the presence of DCBQ, which diffuses away from PSII.^[10]^ The energy level of electrons on the Q_B_ site (−0.06 V vs. NHE) in DET process or DCBQ (+0.315 V vs. NHE) in MET are much lower than the CB position of the carbon nitride (−1.18 V vs. NHE for MCN and −0.94 V vs. NHE for b-CN). Given these values, the electron injection from the Q_B_ sites of PSII or DCBQ to the CB of MCN or b-CN is energetically unfavorable. Instead, the electrons from Q_B_ sites can feasibly combine with the photoinduced holes on the VB of MCN or b-CN, leaving separated electrons on the CB of MCN or b-CN. This results in a Z-scheme structure, resembling a previously reported electron transport mechanism.^[11]^


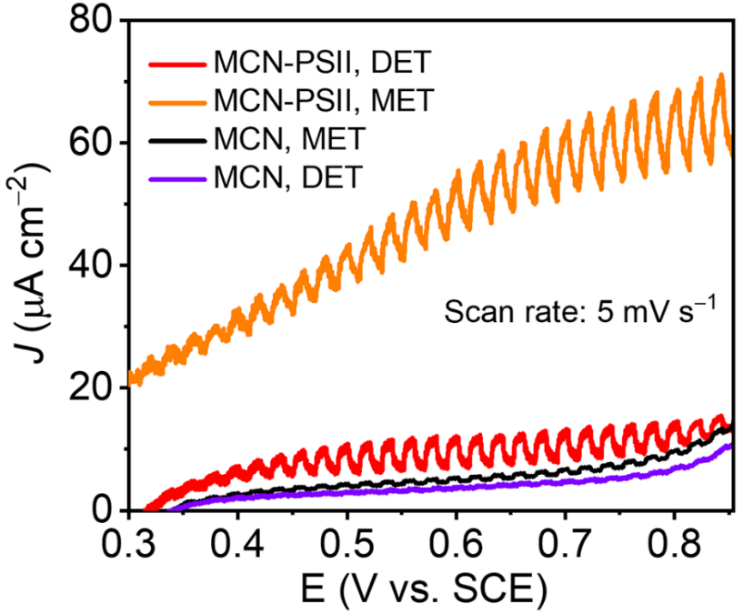


**Figure S30** LSV curves of the 0.196 cm^2^ photoanodes in a three-electrode system. Representative linear sweep voltammetry (LSV) curves were recorded on MCN and MCN-PSII electrodes from 0.30-1.0 V vs. SCE under chopped solar light irradiation with a 2 s on/off interval and 5 mV s^−1^ scan rate. MCN displayed a marginal DET photocurrent response over the entire potential range. This photoresponse can be likely assigned to capacitive charging of the porous electrode, or the well-known charge accumulation of carbon nitride^[12]^ during the transient LSV scans. After wiring with PSII, the MCN-PSII hybrids showed a negatively shifted open circuit potential and significantly enhanced DET photocurrent from 0.30-0.50 V vs. SCE, indicating an enhanced water oxidation kinetics and photoelectric response. After adding 1 mM DCBQ as diffusional electron mediator to the PSII buffer solution, the MET photocurrent density of MCN-PSII electrodes was further enhanced, while the bare MCN photocurrent only showed a slight increase. Based on LSV curves, the initially detected open circuit potential values of photoanodes, 0.40 and 0.60 V vs. SCE were selected as the E during PEC studies.


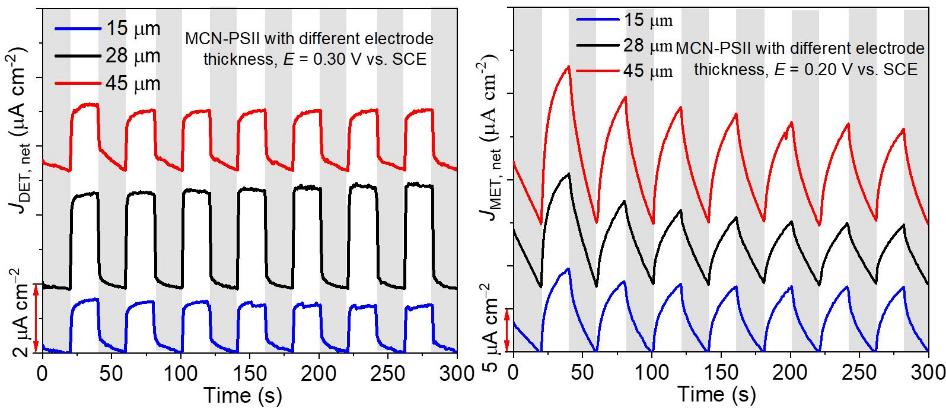


**a**

**b**

**Figure S31** PEC tests of 0.196 cm^2^ MCN-PSII photoanodes of different electrode thicknesses. (a) J_DET, net_ of MCN-PSII photoanode. (b) J_MET, net_ (with 1 mM DCBQ) of MCN-PSII photoanode. The tests were conducted in air, under chopped light illumination (AM 1.5G, 80 mW cm^−2^). Current traces are processed by dark current subtraction. The grey-shaded areas represent dark current intervals. The optimal electrode thickness for J_DET_ is about 28 μm. J_MET_ increased with higher electrode thickness from 15-45 μm, which allowed for more PSII anchoring. However, a thicker layer (> 40 µm) can lead to saturation in photocurrent due to higher resistance, limited mass transfer and light-shielding.^[13]^ Hence, we chose 28 μm as the optimal electrode thickness. The DET photocurrent requires a shorter time to reach equilibrium, whereas the MET photoresponse is related to the role of DCBQ. The latter can convey more photogenerated electrons from the Q_B_ of PSII to MCN or b-CN electrodes, taking longer to reach a steady-state photocurrent.


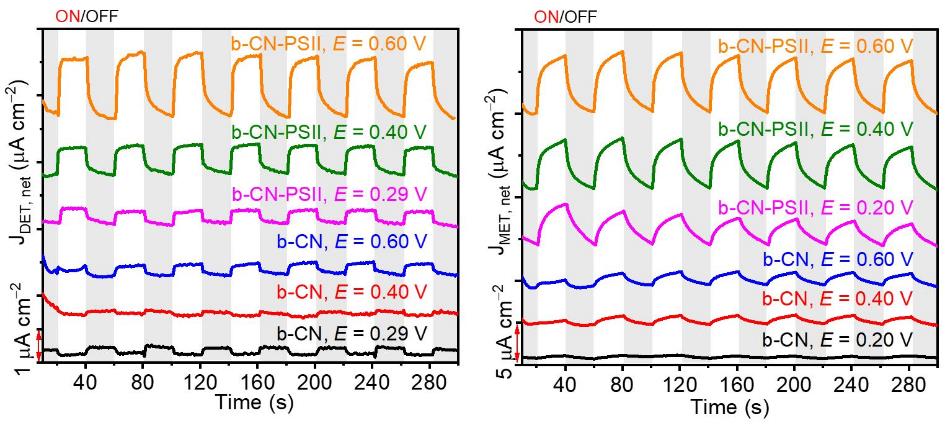


**a**

**b**

**Figure S32** PEC tests of 0.196 cm^2^ b-CN and b-CN-PSII photoanodes at different E vs. SCE. (a) J_DET, net_. (b) J_MET, net_ (with 1 mM DCBQ) tests in air, under chopped light illumination (AM 1.5G, 80 mW cm^−2^). Current traces are processed by dark current subtraction. The grey-shaded areas represent dark current intervals.


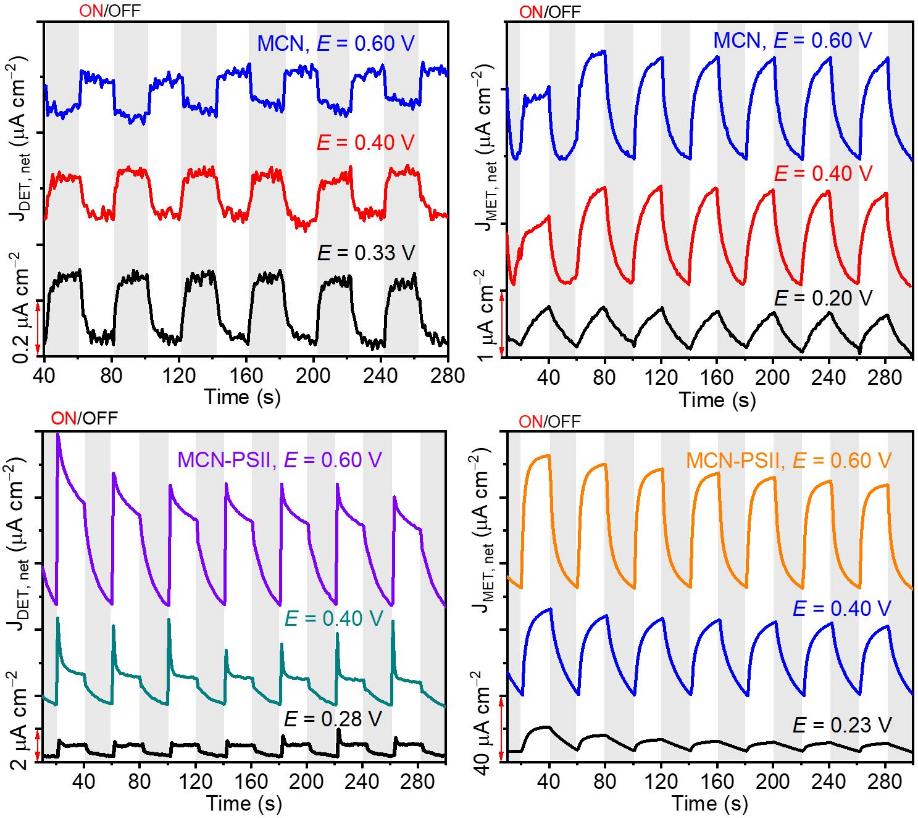


**a**

**b**

**c**

**d**

**Figure S33** PEC tests of 0.196 cm^2^ MCN and MCN-PSII photoanodes at different E vs. SCE. (a) J_DET, net_ of MCN photoanode. (b) J_MET, net_ (with 1 mM DCBQ) of MCN photoanode. (c) J_DET, net_ of MCN-PSII photoanode. (d) J_MET, net_ (with 1 mM DCBQ) of MCN-PSII photoanode. The tests were conducted in air, under chopped light illumination (AM 1.5G, 80 mW cm^−2^). Current traces are processed by dark current subtraction. The grey-shaded areas represent dark current intervals.


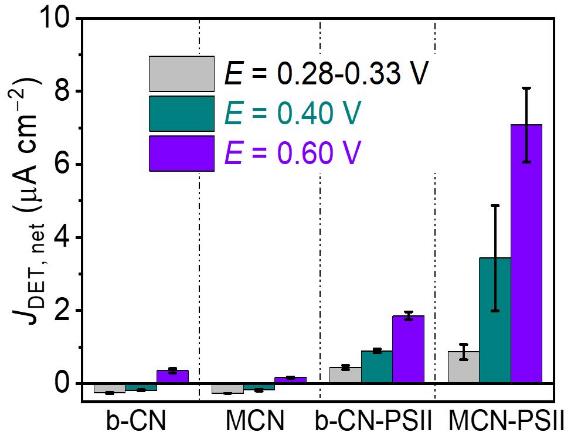


**Figure S34** Average J_DET, net_ values at different E vs. SCE. Data were obtained based on Figures S32, S33.


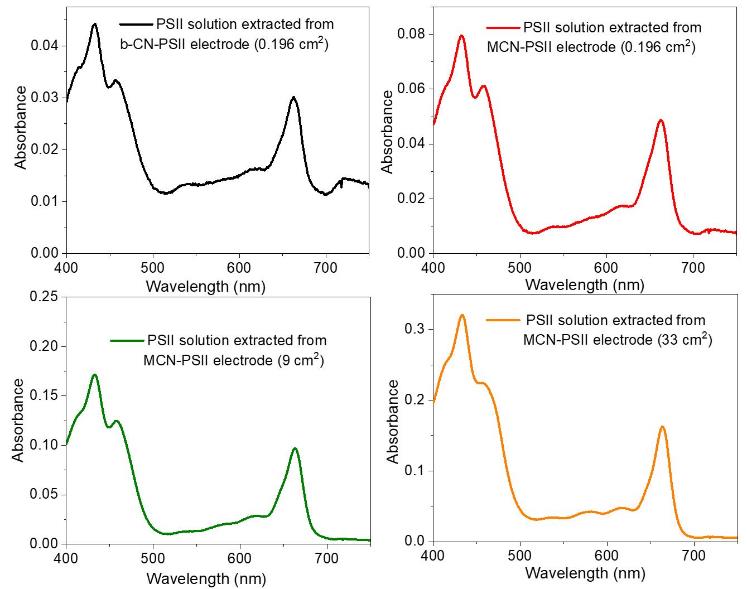


**b**

**a**

**d**

**c**

**Figure S****35** Representative UV-vis spectra of PSII solution extracted from different electrodes. (a) Extract from 0.196 cm^2^ b-CN-PSII electrode. (b) Extract from 0.196 cm^2^ MCN-PSII electrode. (c) Extract from 9 cm^2^ MCN-PSII electrode. (d) Extract from 33 cm^2^ MCN-PSII electrode. PSII dimers were extracted by dissolving in acetone/water solution; Chl *a* concentration was calculated by UV-vis spectra, based on Equation 1 (see Methods).


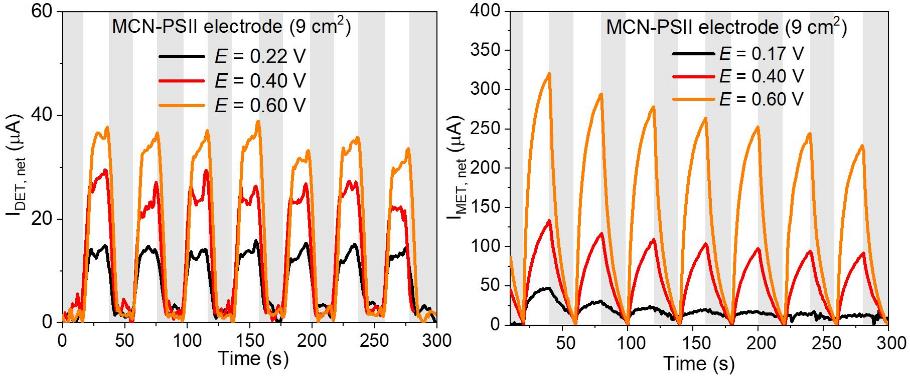


**b**

**a**

**Figure S36** PEC tests of 9 cm^2^ MCN-PSII electrodes at different E vs. SCE. (a) DET and (b) MET net photocurrents in air, under chopped light illumination (AM 1.5G, 80 mW cm^−2^). Current traces are processed by dark current subtraction. The grey-shaded areas represent dark current intervals.


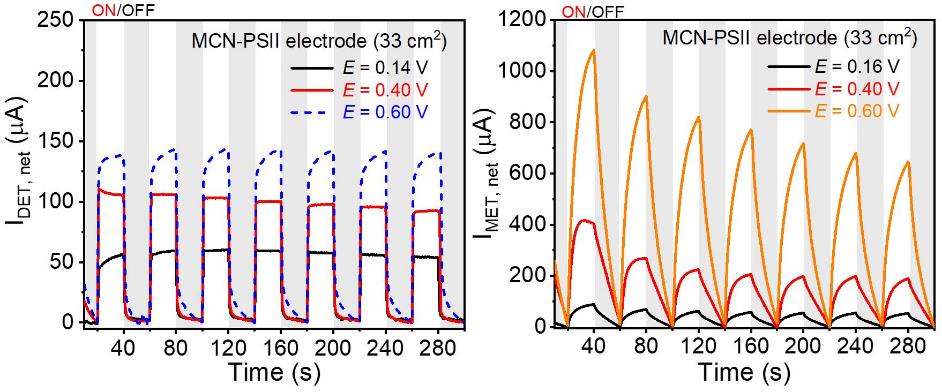


**b**

**a**

**Figure S37** PEC tests of 33 cm^2^ MCN-PSII electrodes at different E vs. SCE. (a) DET and (b) MET net photocurrents in air, under chopped light illumination (AM 1.5G, 80 mW cm^−2^). Current traces are processed by dark current subtraction. The grey-shaded areas represent dark current intervals. As shown in Figures S35, S36, The 9 and 33 cm^2^ MCN-PSII electrodes delivered net MET photocurrents (I_MET, net_) of 295.7 and 907.8 µA at 0.60 V vs. SCE, which correspond to J_MET, net_ of 32.9 and 27.5 µA cm^−2^, respectively. The lower J_MET, net_ compared to 0.196 cm^2^ electrodes (77.4±2.6 μA cm^−2^ at 0.6 V vs. SCE) may arise from the increased resistive losses and inhomogeneous potential distribution over larger electrode areas,^[14]^ leading to decreased photocurrents.


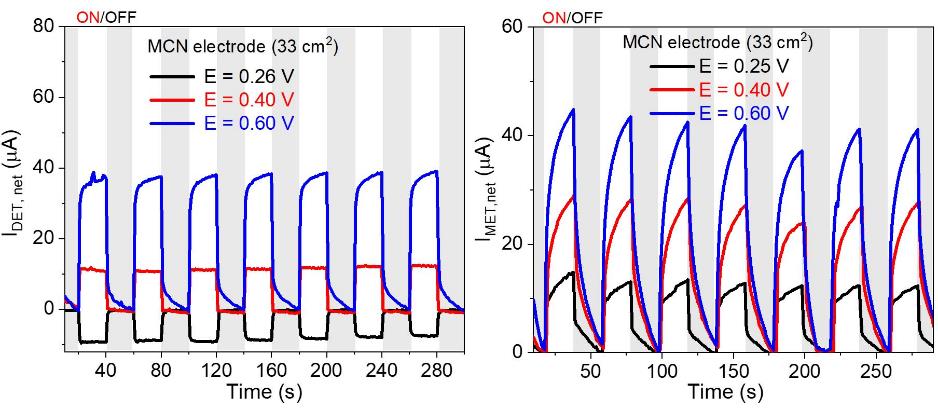


**b**

**a**

**Figure S38** PEC tests of 33 cm^2^ MCN electrodes at different E vs. SCE. (a) DET net photocurrent and (b) MET net photocurrent in air, under chopped light illumination (AM 1.5G, 80 mW cm^−2^). Current traces are processed by dark current subtraction. The grey-shaded areas represent dark current intervals.


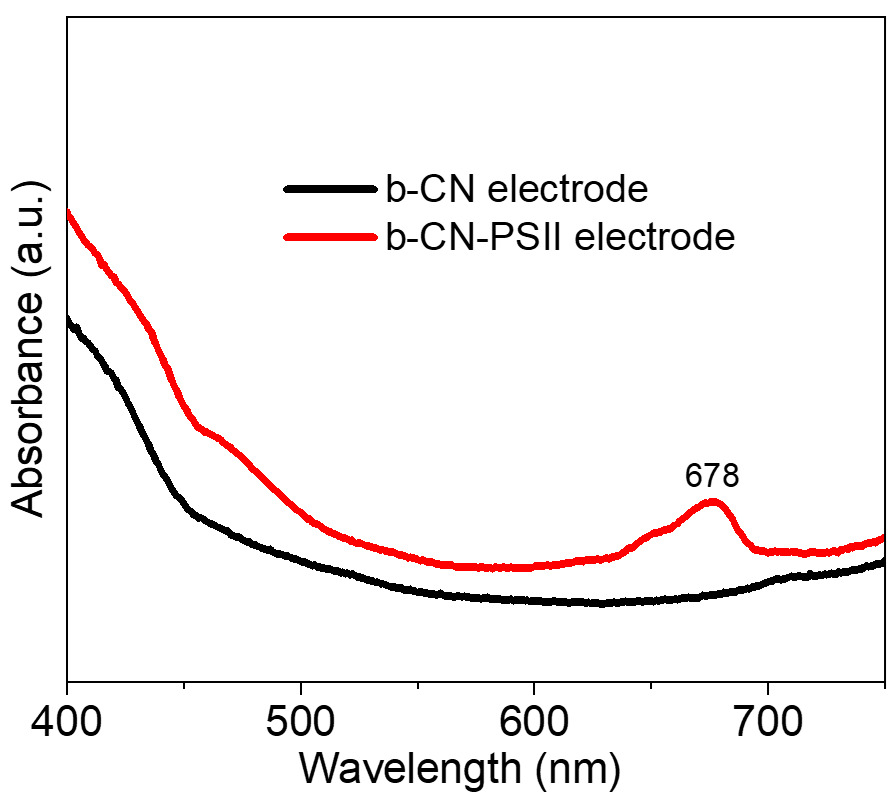


**Figure S39** UV-vis spectra of 0.196 cm^2^ b-CN and b-CN-PSII electrodes. Compared with b-CN, the b-CN-PSII electrode displayed characteristic absorption peaks but with a relatively weak enhancement due to its lower PSII loading.


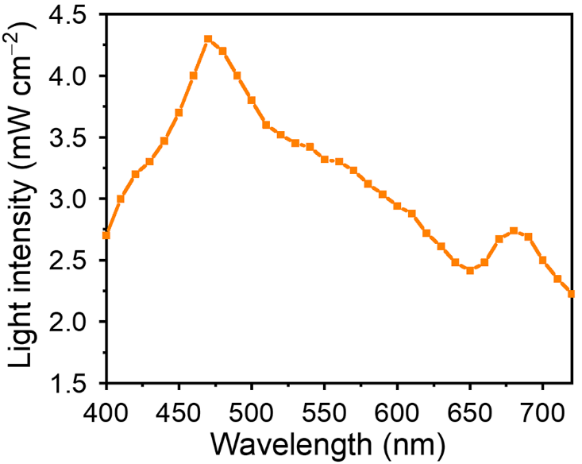


**Figure S40** Light intensity as a function of wavelength during monochromatic light irradiation.


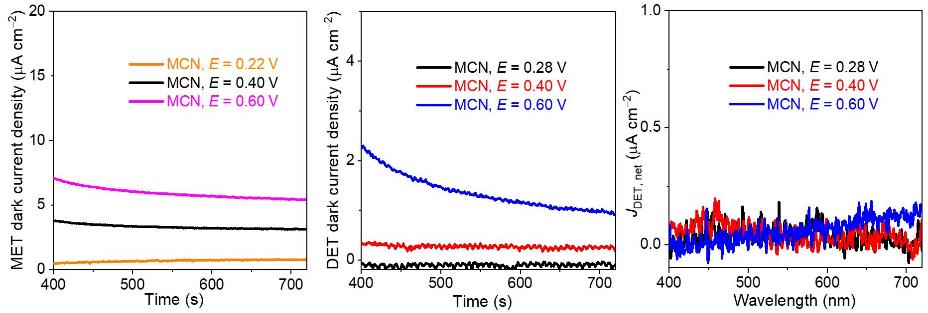


**b**

**a**

**c**

**Figure S41** Dark current and photocurrent action spectra of MCN electrodes at different E vs. SCE. (a) MET and (b) DET current responses of 0.196 cm^2^ MCN electrodes under dark. (c) DET photocurrent action spectra of 0.196 cm^2^ MCN electrodes under continuous monochromatic light irradiation from 400 to 720 nm. The spectra depict J_DET,_ _net_ after subtracting the corresponding dark currents in (b).


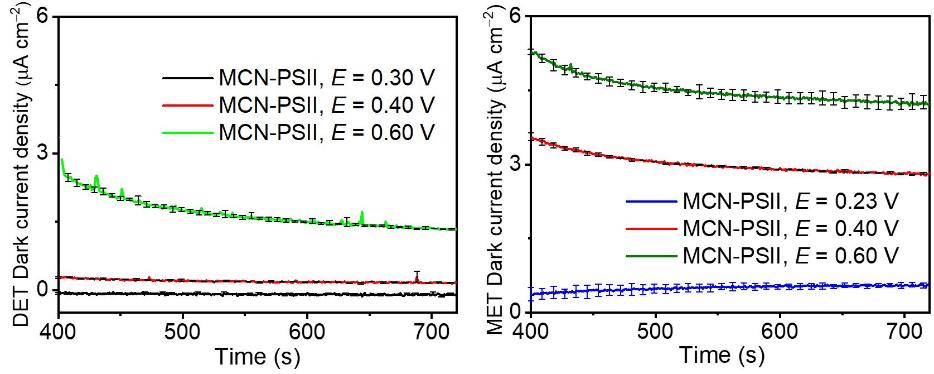


**b**

**a**

**Figure S42** Dark current spectra of MCN-PSII electrodes at different E vs. SCE. (a) DET and (b) MET current responses of 0.196 cm^2^ MCN-PSII electrodes under dark.


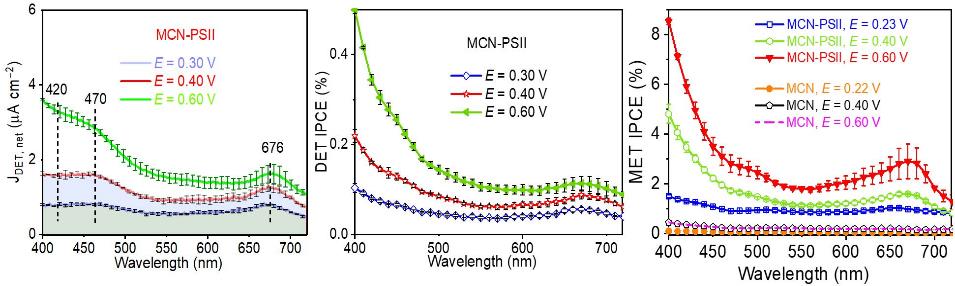


**b**

**a**

**c**

**Figure S43** Photocurrent action spectra of 0.196 cm^2^ MCN-PSII photoanodes at different E vs. SCE. (a) DET photocurrent spectra under continuous monochromatic light (400-720 nm) irradiation. The spectra reflect J_DET,_ _net_ after subtracting corresponding dark current spectra (Figure S42a). (b) Calculated IPCE for the DET processes. (c) IPCE for the MET processes at different E vs. SCE, calculated based on Figure 3e.


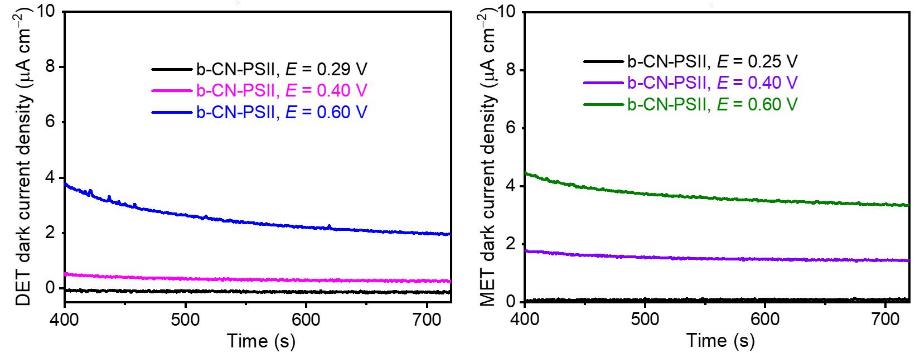


**b**

**a**

**Figure S44** Dark current spectra of b-CN-PSII electrodes at different E vs. SCE. (a) DET and (b) MET current responses of 0.196 cm^2^ b-CN-PSII electrodes under dark.


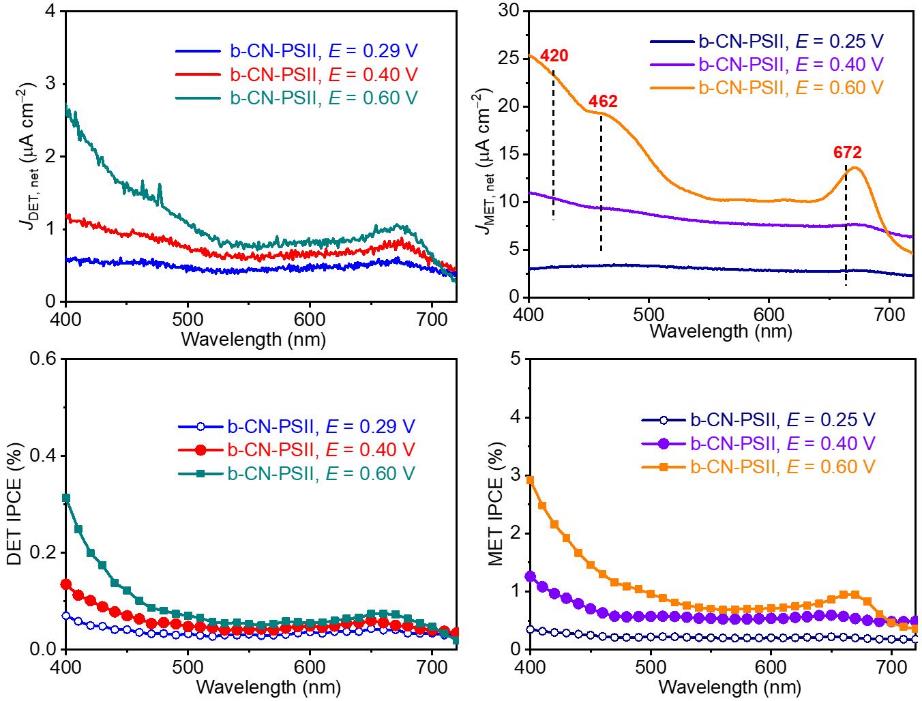


**b**

**a**

**d**

**c**

**Figure S45** DET and MET photocurrent action spectra of 0.196 cm^2^ b-CN-PSII electrodes to single-wavelength irradiation at different E vs. SCE. (a) DET and (b) MET photocurrent responses under continuous monochromatic light irradiation, scanned from 400 to 720 nm. The spectra reflect J_DET,_ _net_ after subtracting corresponding dark current spectra (Figure S44). (c) DET IPCE and (d) MET IPCE.

**Figure S46** H_2_ evolved at the Pt counter electrode during MET PEC tests at E = 0.6 V vs. SCE, when testing 33 cm^2^ MCN-PSII or MCN photoanodes in a three-electrode system during 12 h/6 h on-off irradiation.


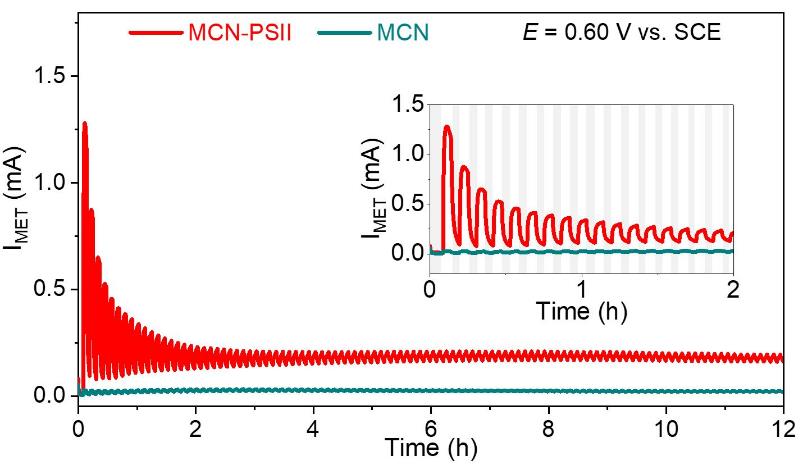


**Figure S47** Examples of MET photocurrents for 33 cm^2^ MCN-PSII and MCN electrodes during 200 s on-off irradiation cycles. The grey-shaded areas represent dark current intervals.


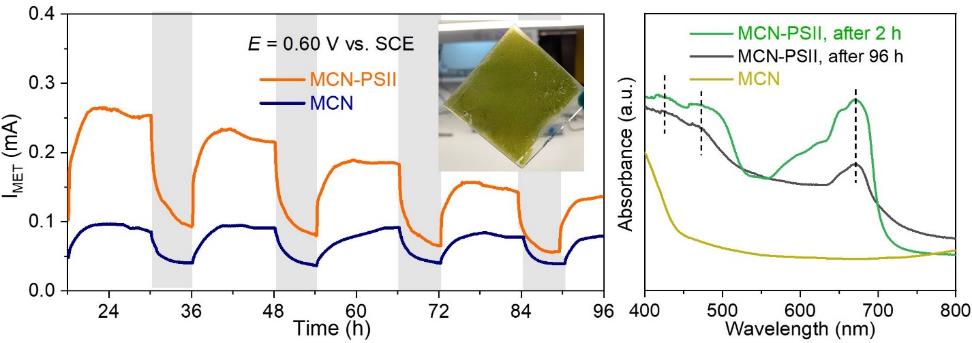


**b**

**a**

**Figure S48** PEC performance of large-area electrodes under N_2_ atmosphere. (a) MET photocurrents of 33 cm^2^ MCN-PSII and MCN photoanodes from 18 h to 96 h over 12-h light/6-h dark cycle (AM 1.5 G, 80 mW cm^2^). Grey areas indicate dark periods. Inset show the photograph of the 33 cm^2^ MCN-PSII electrode after the 4-day test. (b) UV-vis spectra comparison of 33 cm^2^ MCN-PSII electrodes after 2 h and 96 h PEC tests.


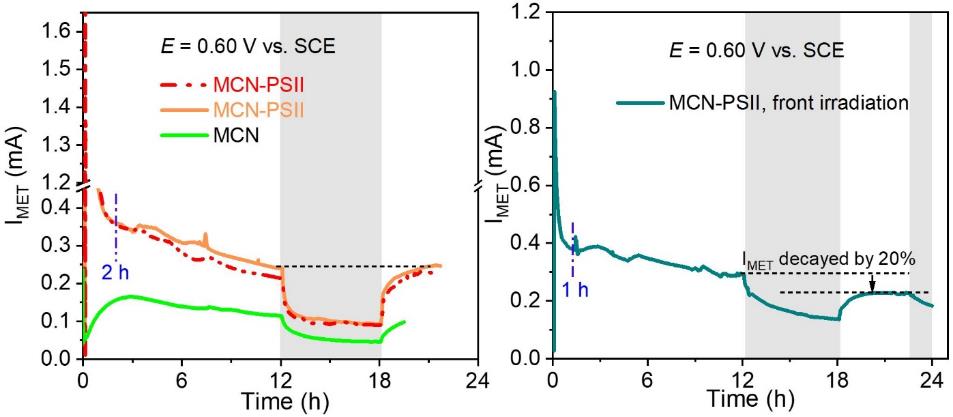


**a**

**b**

**Figure S49** Examples of MET photocurrents for 33 cm^2^ MCN-PSII and MCN electrodes during 12-h/6-h on-off irradiation. (a) Back irradiation (all electrodes are illuminated from the back unless otherwise specified). (b) Front irradiation. The grey-shaded area represents the dark period. The front-irradiated 33 cm^2^ MCN-PSII electrode displayed a slightly lower initial photocurrent and a faster initial photocurrent decay. This preferential orientation may be caused by PSII partly blocking the light in a front-irradiated electrode, whereas MCN absorbs UV-light, acting as a shield against PSII photodegradation.

**a**

**b**

**c**

**d**

**Figure S50** Detection of H_2_O_2_ concentrations in the electrolyte after PEC tests, using the colorimetric N,N-diethyl-p-phenylenediamine (DPD) method. (a) UV-vis absorption spectrum for H_2_O_2_ detection of the solution from a 33 cm^2^ MCN-PSII electrode, after a 24-h test. (b) MCN (33 cm^2^) after a 48-h test. (c) b-CN-PSII (33 cm^2^) after a 6-day test. (d) b-CN (33 cm^2^) after the 4-day test. The amounts of H_2_O_2_ (0.12-0.34 μmol) are negligible compared to the 13-20 μmol O_2_ obtained during the first 5 h for MCN-PSII and b-CN-PSII electrodes (Figures 4c, S51b), suggesting that incomplete water oxidation is not a significant side reaction. Similarly, only 0.28 and 0.27 μmol H_2_O_2_ are detected in the electrolyte of MCN and b-CN.


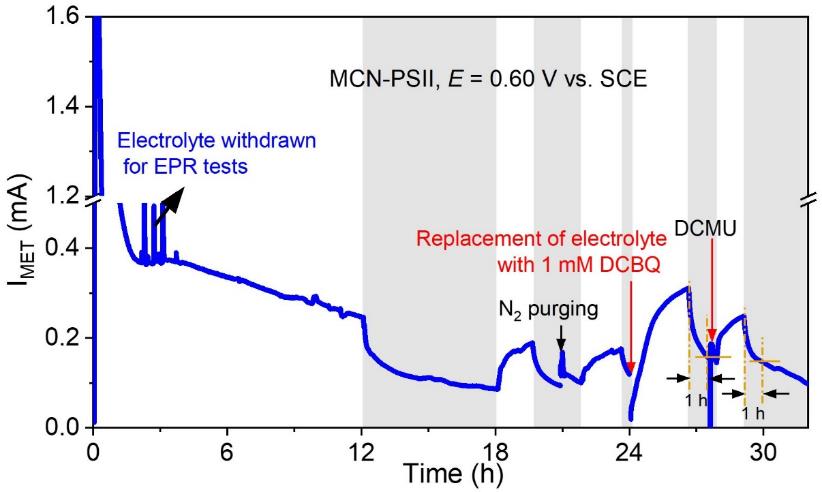


**Figure S51** Factors influencing the MET photocurrent of a 33 cm^2^ MCN-PSII electrode. The test was performed in air between 0-20 h and after 24 h, with N_2_ purging conducted between 20-24 h. A fresh electrolyte solution with 1 mM DCBQ was used after 24 h, and 2 mM DCMU was added after ~27 h. Grey areas indicate dark periods. An enhanced photocurrent response was observed when replacing the electrolyte with a fresh solution, indicating that DCBQ degradation contributes to the gradual decrease in PEC performance.


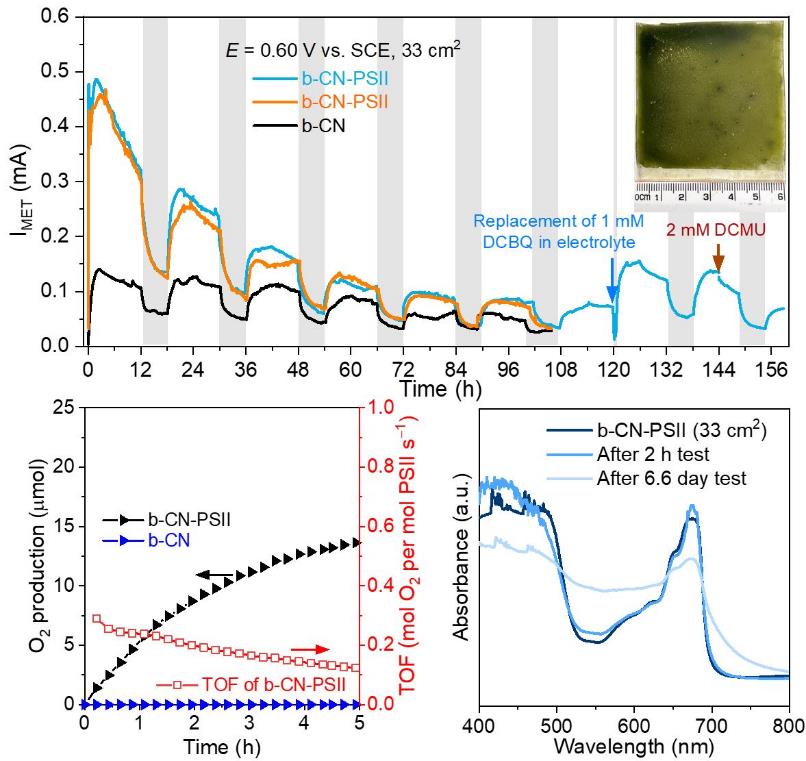


**a**

**b**

**c**

**Figure S52** PEC tests of b-CN-PSII and b-CN electrodes. (a) MET photocurrents of 33 cm^2^ b-CN-PSII and b-CN electrodes. Grey areas indicate dark periods. Fresh electrolyte with 1 mM DCBQ was added after 120 h. The photocurrent decreased when introducing DCMU after 144 h. Insert shows the electrode after the 6.6 days (158 h) test. (b) O_2_ evolution and corresponding TOF during the first 5 h of irradiation of b-CN-PSII (Chl *a* loading: 0.19 mg) and b-CN photoanodes in (a). (c) UV-vis diffuse reflectance spectra of b-CN-PSII before, during and after 6.6-day MET PEC tests at 0.6 V vs. SCE. Spectra before and after a 2 h test are recorded on the same electrode. The spectrum after 6.6 days corresponds to the sample tested in (a). The b-CN-PSII photocurrent increased when adding fresh electrolyte with 1 mM DCBQ (a), indicating that DCBQ degradation is partly accountable for the long-term photocurrent decay. UV-vis absorption peaks of Chl *a* remained visible after 6.6 days (c).

**a**

**b**

**c**

**Figure S53** EPR spectra detected in the electrolyte solution during the MET PEC tests of 33 cm^2^ b-CN-PSII in Figure S51. (a) EPR spectra of the electrolyte solution. (b) EPR spectra with additional TEMP for capturing ^1^O_2_. (c) EPR spectra with DMPO for •OH detection. DCBQ peaks are hardly detected after 20 and 120 h (a), whereas only weak peaks of the DCBQ radical were identified after 20 and 120 h with TEMP as a capturing agent (b). No •OH and ^1^O_2_ peaks emerged when using either TEMP or DMPO as capturing agents, suggesting that these ROS were not produced in the electrolyte of b-CN-PSII.


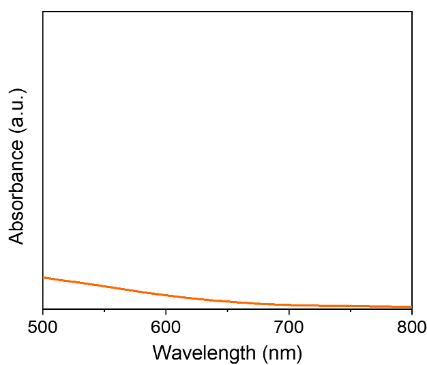


**Figure S54** UV-vis diffuse reflectance spectrum. Acetone/water were used to extract any PSII from the centrifuged reaction electrolyte after the 6.6-day test on b-CN-PSII (Figure S51a), and no UV-vis absorption peaks of PSII were found. This indicates a negligible PSII detachment during operation.

**a**

**b**

**Figure S55** X-ray absorption near-edge structure (XANES) spectra of a CNT film. (a) C K-edge. (b) O K-edge. XANES spectra of the commercial CNT film suggests the existence of C and O functional groups. The C K-edge spectrum shows the transitions of C−C π* excitation (sp^2^ C), C−O or C=O (sp^3^ C), and C−C σ*, respectively.^[15]^ O K-edge spectrum indicates the existence of C=O or O−C=O, OH, and C−O σ*.^[16]^

**b**

**a**


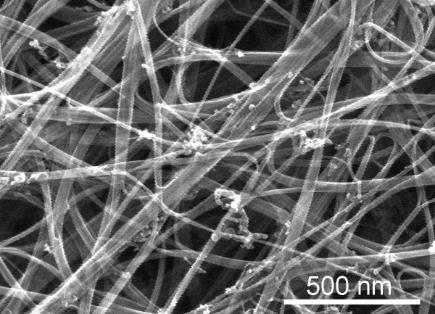

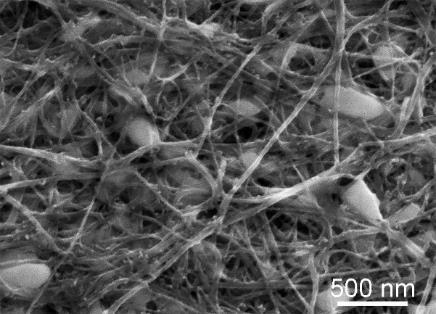


**Figure S56 SEM images.** (a) CNT film. (b) CNT-BOD film. SEM image displays the well-assembled CNT with sufficient large gaps on CNT film to wrap BOD enzymes in CNT-BOD electrode.


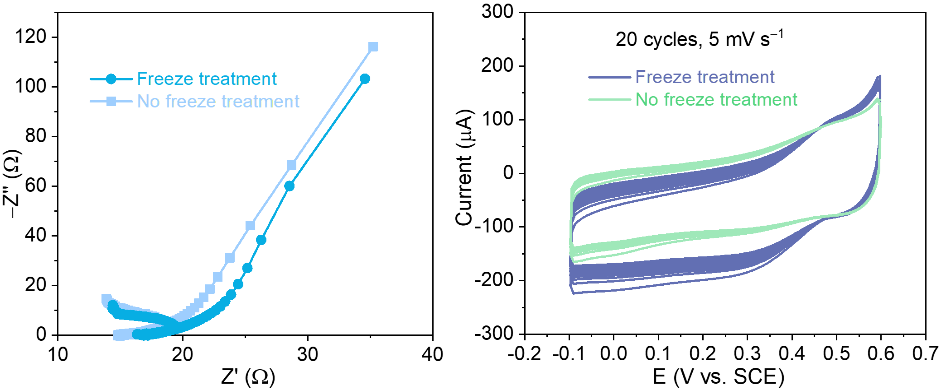


**a**

**b**

**Figure S57** The effect of freeze treatment on CNT-BOD cathodes (2×2 cm^2^). (a) EIS spectra with or without freeze treatment. (b) CV curves recorded from 0.6 V to ‒0.1 V vs. SCE in PSII buffer solution under air. Scan rate: 5 mV s^−1^.


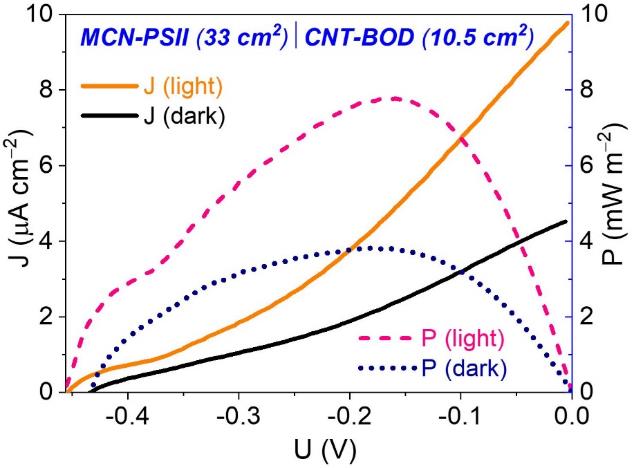


**Figure S58** *J*-*U* curves. The curves were recorded between ‒0.55 V and 0 V, at 1 mV s^−1^, for the MCN-PSII (33 cm^2^)|CNT-BOD (10.5 cm^2^) two-electrode BPV device.


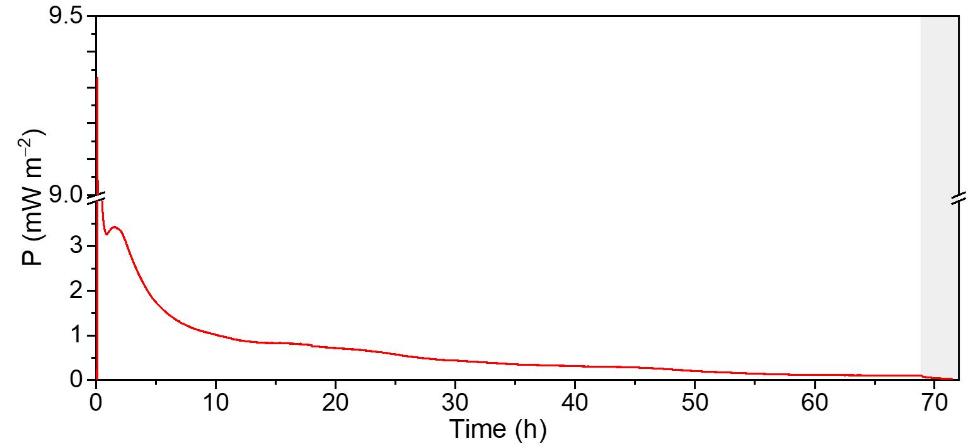


**Figure S59** Power output of an MCN-PSII (9 cm^2^)|CNT-BOD (4 cm^2^) cell operating over 69 h illumination. The grey-shaded area represents the dark period.


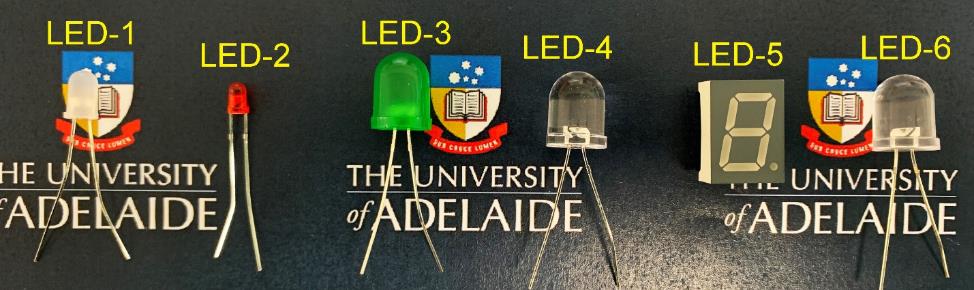


**Figure S60** Photographs of different LED lights and LED display involved in BPV tests. The specifications of these LED electronics are found in Table S4. (*The University of Adelaide name and logo are reproduced with permission from the University of Adelaide.*)

**
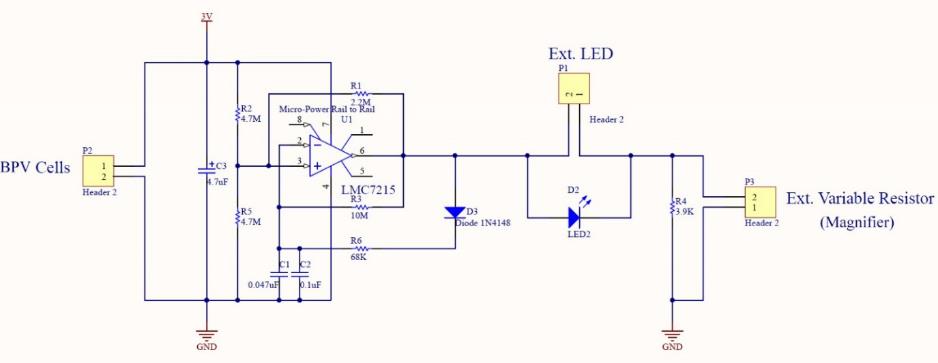
**

**Figure S61** The electronic circuit diagram for powering LEDs. The diagram is adapted from the Flasher/Blinker Circuits-Ultra Low Current 3V LED Flasher on the website of Discover Circuits.com, US.

**
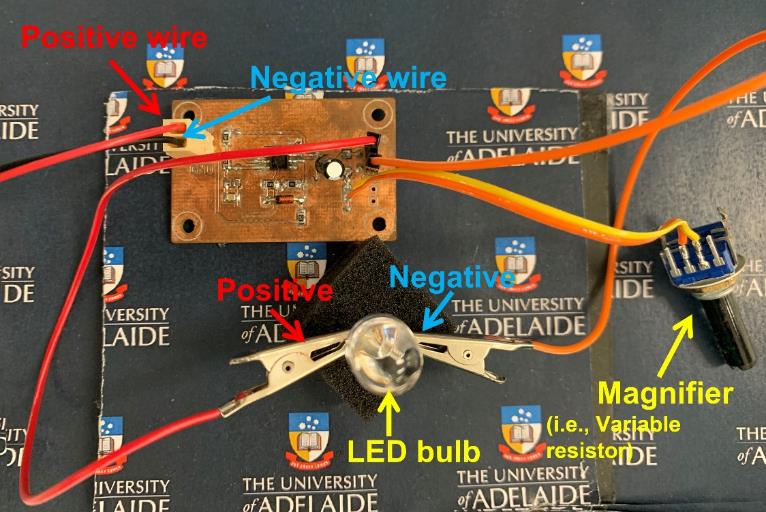
**

**Figure S62** Photograph of the ultra-low current LED flasher circuit board. LEDs 1, 3, 4, 5 or 6 were connected to the positive and negative clips for flashing tests. LED-2 was directly welded on the electric board for flashing tests. The circuit is also equipped with a variable resistor (magnifier), which can increase the power output from the BPV cell by changing the circuit resistance, to enhance the flashing brightness of the LED. (*The University of Adelaide name and logo are reproduced with permission from the University of Adelaide.*)


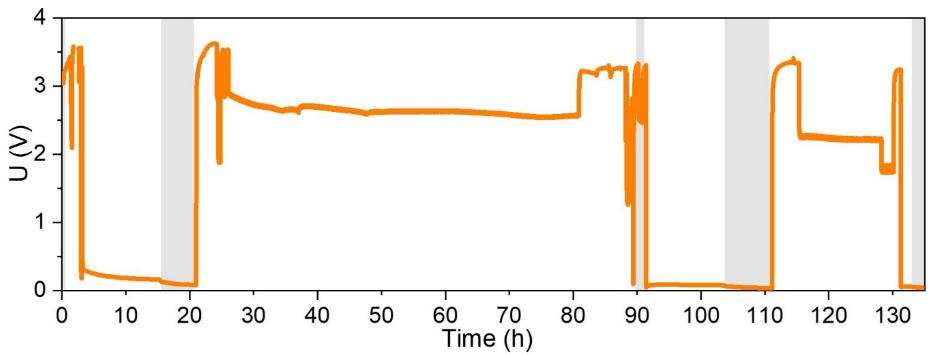


**Figure S63** Voltage output of 8 serially connected MCN-PSII (9 cm^2^)|CNT-BOD (4 cm^2^) BPV cells for 135 h. Grey areas indicate dark periods. Different LED lights, LED display and resistors were connected at different times; details are provided in Figures 5 and S63.


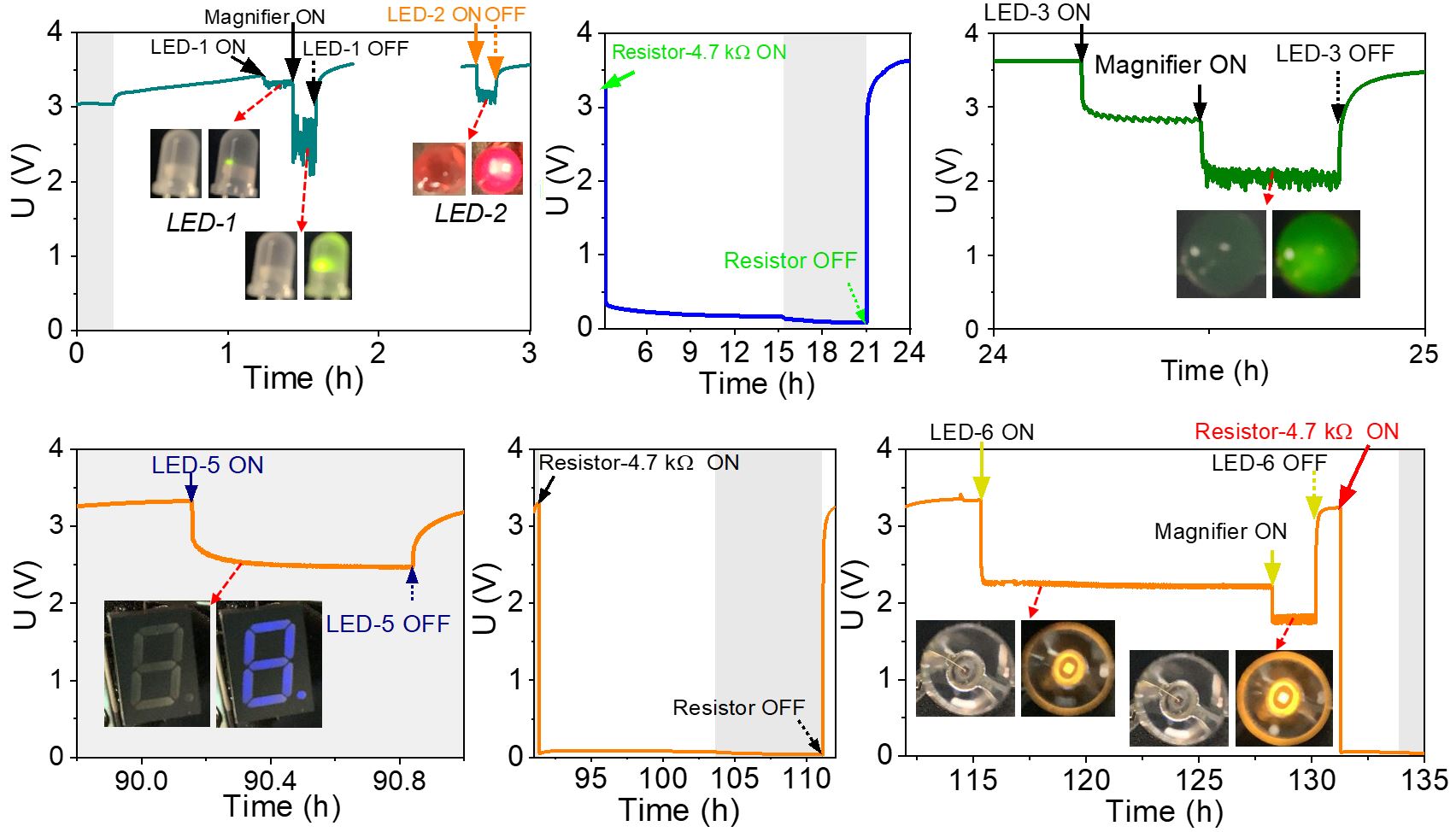


**a**

**b**

**f**

**e**

**c**

**d**

**Figure S64** Details of the BPV test in Figure S62. (a-f) Voltage output from 8 tandem MCN-PSII (9 cm^2^)|CNT-BOD (4 cm^2^) PEC cells connected in series, with different LED lights, displays and resistors (AM 1.5 G, 100 mW cm^-2^ irradiation). The sealed photoanode compartment remained under N_2_ atmosphere, while the cathode compartment was exposed to air. Grey areas indicate dark periods.


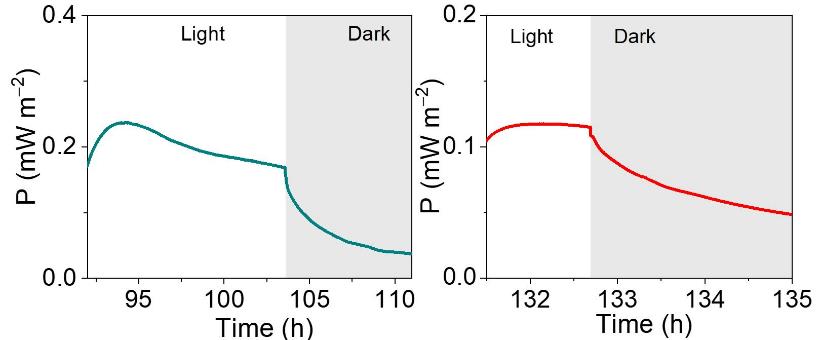


**a**

**b**

**Figure S65** Power output during the (a) 92-111 h interval and (b) 131.5-135 h interval under light and dark.


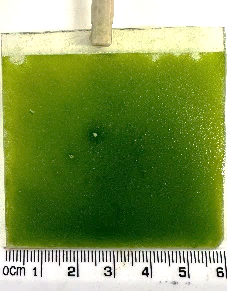

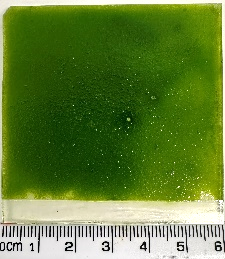

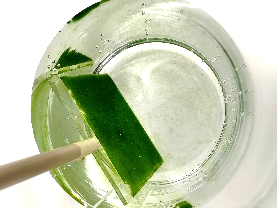

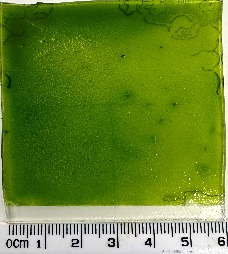

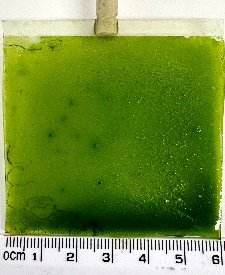

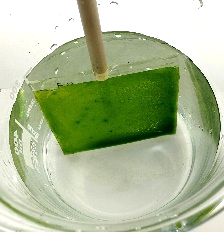


**c**

**b**

**a**

**f**

**e**

**d**

**Figure S66** Photos of 33 cm^2^ MCN-PSII and b-CN-PSII electrodes. (a) MCN-PSII. (b) Dipping of MCN-PSII into PSII buffer solution. (c) MCN-PSII after dipping. (d) b-CN-PSII. (e) Dipping of b-CN-PSII into PSII buffer solution. (f) b-CN-PSII after dipping.

**Table S1 Photocurrent data and PSII loading of different electrodes.** All applied potentials are given vs. SCE.

| **Electrode** | **Loading amount**  **(Chl *a*)** | **Loading amount (PSII)** | **J_DET, net,_**  **0.14-0.33 V**  (µA cm^−2^) | **J_DET, net,_**  **0.40 V**  (µA cm^−2^) | **J_DET, net,_**  **0.60 V**  (µA cm^−2^) | **J_MET, net,_**  **0.16-0.25 V**  (µA cm^−2^) | **J_MET, net,_**  **0.40 V**  (µA cm^−2^) | **J_MET, net,_**  **0.60 V**  (µA cm^−2^) |
| --- | --- | --- | --- | --- | --- | --- | --- | --- |
| **b-CN**  **(0.196 cm^2^)** | - | - | −0.3, 0.29 V | −0.2 | 0.4±0.1 | 0.6±0.1,  0.20 V | 1.1±0.1 | 1.7±0.1 |
| **b-CN-PSII (0.196 cm^2^)** | 0.9±0.2 μg | 29.7±6.4 pmol | 0.4, 0.29 V | 0.9±0.1 | 1.9±0.1 | 4.0±1.2,  0.20 V | 5.8±0.4 | 7.2±0.3 |
| **MCN**  **(0.196 cm^2^)** | - | - | −0.3,  0.33 V | −0.2 | 0.2 | 0.6,  0.20 V | 1.5 | 1.6±0.1 |
| **MCN**  **(33 cm^2^)** | - | - | −0.3,  0.26 V | 0.3 | 1.2 | 0.40,  0.25 V | 0.9 | 1.3  (I_MET_, _net_  43.6 µA) |
| **MCN-PSII**  **(0.196 cm^2^)** | 2.6±0.4 μg | 81.9±13.8 pmol | 0.9±0.2,  0.28 V | 3.4±1.4 | 7.1±1.0 | 12.4±2.3,  0.23 V | 50.4±2.0 | 77.4±2.6 |
| **MCN-PSII**  **(9 cm^2^)** | 0.09±0.03 mg | 2.88±0.96 nmol | 1.7,  0.22 V | 3.0 | 4.1 | 3.5,  0.17 V | 13.1 | 32.9  (I_MET_, _net_  295.7 µA) |
| **MCN-PSII**  **(33 cm^2^)** | 0.24±0.06 mg | 7.73±1.81 nmol | 1.8,  0.14 V | 3.2 | 4.3 | 2.2,  0.16 V | 8.2 | 27.5  (I_MET_, _net_  907.8 µA) |

**Table S2 PEC performance of reported PSII electrodes.**

| **Electrode** | **PSII source** | **Light source** | **Method for electrode fabrication** | **Area (cm^2^)** | **Applied potential (V)** | **J_DET_**  **(µA**  **cm^−2^)** | **J_MET_**  **(µA**  **cm^−2^, with DCBQ as mediator, unless specified)** | **Total MET current (µA)** | **Ref.** |
| --- | --- | --- | --- | --- | --- | --- | --- | --- | --- |
| IO-TiO_2_\|dpp\|P_Os_-PSII | T. elongatus | λ>420nm, 100mWcm^–2^ | Deposition | 0.25 | -0.2 V- 0.7 V vs. SHE | - | 70 - 80 μAcm^−2^  (Poly(1-vinylimidazole-co-allylamine)-Os(bpy)_2_Cl, i.e., P_os_ as mediator) | 17.5-20 | ^[10]^ |
| ITO-(PEI/PSII)_2_ | Spinach | 550-800 nm (P_680nm_=10 mW cm^−2^) | Dip coating | 0.42 | 0.25 V vs. SCE | 0.02 | - | 0.0084 | ^[17]^ |
| ITO-(PEI- rGO/PSII)_3_ | Spinach | 550-800 nm (P_680nm_=10 mW cm^−2^) | Dip coating | 0.42 | 0.25 V vs. SCE | 0.04 | - | 0.017 | ^[17]^ |
| PSII on IO- graphene | T. elongatus | 685 nm,  10 mW cm^−2^ | Dropcasting | 0.25 | 0.50 V vs. SHE | 0.30 ±0.01 | 12.3±0.4 | 3.08±0.09 | ^[18]^ |
| PSII on ITO-SA 750 nm | T. elongatus | 685 nm,  10 mW cm^−2^ | Dropcasting | 0.25 | 0.50 V vs. SHE | 1.42±0.06 | 40.2±0.9 | 10.1±0.2 | ^[18]^ |
| PSII/(ITO)_15_/Au | Spinach | 550-800 nm,  (P_680nm_=10 mW cm^−2^) | Dropcasting | 0.78 | 0.25 V vs. SCE | 2.36±0.07 | 39.0±1.7 | 30.4±1.3 | ^[19]^ |
| PSII/(TiO_2_)_5_- NTs/ITO | Spinach | 550-800 nm (10 mW cm^−2^) | Dropcasting | 0.78 | 0.25 V vs. SCE | 1.3 | 10.6 | 8.27 | ^[20]^ |
| PSII/NCNT | Spinach | 1 sun (λ > 450 nm),  78 mW cm^−2^ | Centrifuge | 0.07 | 0.30 V vs. Ag/AgCl | 1.25 | - | - | ^[22]^ |
| C-PEI-PSII | Spinach | 670 nm,  2.67 mW m^−2^ | Dip coating | 0.196 | 0.17 V vs. SCE | 0.81±0.21 | 15.8±0.4 | 3.09±  0.08 | ^[22]^ |
| C-PEI-PSII | Spinach | 1 sun, 350-800 nm,  80 mW cm^−2^ | Dip coating | 0.196 | 0.17 V vs. SCE | 1.78±0.14 | 18.01±0.60 | 3.53±  0.12 | ^[22]^ |
| PSII-modified ITO | T. elongatus | λ > 685 nm, 20 mW cm^−2^ | Spray coating | 1 | 0.35 V vs. Ag/AgCl | - | 22.5  (P_os_ as the mediator) | 22.5 | ^[23]^ |
| MCN-PSII | Spinach | 1 sun,  350-800 nm,  80 mW cm^−2^ | Dip coating | 0.196 | 0.60 V vs. SCE | 7.1±1.0 | 77.4±2.6 | 15.2±  0.5 | This work |
| MCN-PSII | Spinach | 676 nm,  2.71 mW m^−2^ | Dip coating | 0.196 | 0.60 V vs. SCE | 1.7±0.2 | 42.9±9.4 | 8.4±  1.8 | This work |
| MCN-PSII | Spinach | 400 nm,  2.7 mW cm^−2^ | Dip coating | 0.196 | 0.60 V vs. SCE | 3.6 | 74.3±1.1 | 14.6±  0.2 | This work |
| MCN-PSII | Spinach | 1 sun, 350-800 nm,  80 mW cm^−2^ | Spray-freeze method | 9 | 0.60 V vs. SCE | 4.1 | 32.9 | 295.7 | This work |
| MCN-PSII | Spinach | 1 sun, 350-800 nm,  80 mW cm^−2^ | Spray-freeze  method | 33 | 0.60 V vs. SCE | 4.3 | 27.5 | 907.8 | This work |

**Table S3 Comparison of MCN-PSII (9 cm^2^)|CNT-BOD (4 cm^2^) PEC cells with previously reported BPV devices using living microorganisms.**

| Photosynthetic microorganism | Exogenous mediator | Anode material | Anode area (cm^2^) | Microorganism status | Method | Maximum P  (mW m^−2^) | Final P (mW m^−2^) | Power output per device (μW) | Testing time | Ref. |
| --- | --- | --- | --- | --- | --- | --- | --- | --- | --- | --- |
| *Photosystem II* | DCBQ | MCN-PSII | 9 | PSII electrode | Spray-freeze method | 9.3 | 0.1 | 8.4 to 0.1  (0-69 h) | 69 h | This work |
| *Photosystem II* | DCBQ | MCN-PSII | 72 (8 devices ×9 cm^2^) | PSII electrode | Spray-freeze method | 2.8  (3.5 h) | 0.1 | 2.5 to 0.1  (3.5 h-132 h) | 5 d | This work |
| *Synechocystis* sp. PCC 6803, wild tpye | Potassium  ferricyanide | 51% In, 32.5% Bi, 16.5% In | 0.0008 | Microorganism suspension in electrolyte | Suspension | 300 | N/A | 0.024 | N/A | ^[24]^ |
| *Synechocystis sp. PCC 6803 – mutant* | Potassium  ferricyanide | 51% In, 32.5% Bi, 16.5% In | 0.0008 | Microorganism suspension in electrolyte | Suspension | 500 | N/A | 0.4 | N/A | ^[25]^ |
| *Synechocystis* sp. PCC 6803 | - | Carbon nanotube-biofilm | 1.36 | Biofilm electrode | Digital printing | ~0.012 | ~0.0098  (~93 h) | ~0.0016 to 0.0013  (0-93 h) | 4 d | ^[25]^ |
| Spinach thylakoids | Potassium  ferricyanide | FTO coated glass-biofilm | 1.08 | Biofilm electrode | Slurry settling | 2500 (≥0.8 V bias provided by a Si PV) | N/A | 270 (≥0.8 V bias provided by a Si PV) | 20 min | ^[26]^ |
| *Synechocystis* sp. PCC 6803 | - | InSnBi alloy-biofilm | 0.0003 | Biofilm electrode | Settling by gravity | 105 | N/A | 0.003 | N/A | ^[27]^ |
| *Nostoc* sp. ATCC 27893 | - | Carbon paper-carbon nanotube-biofilm | 0.025 | Biofilm electrode | Drop coating | 35 | N/A | 0.088 (maximum) | 10 d | ^[28]^ |
| *Nostoc* sp. ATCC 27893 | 1,4-benzoquinone | Carbon paper-carbon nanotube-biofilm | 0.025 | Biofilm electrode | Drop coating | 100 | N/A | 0.25 | N/A | ^[28]^ |
| *Synechocystis* sp. PCC 6803 |  | Poly(3,4-ethylenedioxythiophene):polystyrene sulfonate) treated paper | 3.14 | Microorganism suspension in electrolyte | Suspension | 5.6 | N/A | 1.76 | N/A | ^[29]^ |
| *Chlorella* sp. (UMACC 313) | - | Reduced graphene oxide coated glass-biofilm | 12.25 | Biofilm electrode | Settling by gravity | 0.273 | N/A | 0.33 | N/A | ^[30]^ |
| *Synechocystis* sp. PCC 6803 triple terminal oxidase mutant | Potassium  ferricyanide | ITO coated polyethylene terephthalate | 12.56 | Microorganism suspension in electrolyte | Suspension | 0.183 | N/A | 0.23 | N/A | ^[31]^ |
| *Arthrospira maxima* | - | ITO coated glass-biofilm | 4 | Biofilm electrode | Settling attachment | 0.025 | N/A | 0.01  (maximum) | 8 d | ^[32]^ |
| *Pseudanabaena limnetica* | - | ITO coated polyethylene terephthalate-biofilm | 20 | Biofilm electrode | Settling | 0.024 | N/A | 0.048 | N/A | ^[33]^ |
| *Synechococcus* sp. WH 5701 | - | ITO coated polyethylene terephthalate-biofilm | 12.56 | Biofilm electrode | Settling | 10.3 | N/A | 12.9 | N/A | ^[34]^ |
| *Synechococcus* sp. WH 5701 | - | ITO coated polyethylene terephthalate-biofilm | 110.25 | Biofilm electrode | Settling | 0.05 | 0.03 | 0.55-0.33  (0-32 d) | 32 d | ^[34]^ |
| *Synechocystis* sp. PCC 6803 | Potassium  ferricyanide | ITO coated polyethylene terephthalate | 0.8 | Microorganism suspension in electrolyte | Suspension | 1.2 | N/A | 0.096 | N/A | ^[35]^ |

**Table S4 Parameters for LED electronics used in BPV tests.**

|  | **LED-1**  **(ZD0250)** | **LED-2**  **(ZD0100)** | **LED-3**  **(ZD0205)** | **LED-4**  **(ZD0206)** | **LED-5**  **(ZD1856)** | **LED-6**  **(ZD0226)** |
| --- | --- | --- | --- | --- | --- | --- |
| Color | Green | Red | Green | Green | Blue | Orange |
| Size | 5 mm | 3 mm | 10 mm | 10 mm | 17.5 (H)×12.3 (W)×7 (D) mm | 10 mm |
| Lens or face color | Diffused | Diffused | Diffused | Waterclear | Grey | Waterclear |
| Wavelength | 567 nm | 650 nm | 570 nm | 525 nm | ─ | 600 nm |
| Forward Current (IF) | 20 mA | 15 mA | 20 mA | 20 mA | 10 mA | 20 mA |
| Forward voltage (VF) | 2.2 V | 2.3 V | 2.2 V | 3.1 V | 2.1 V | 2.0 V |
| Luminous intensity (IV) | 10 mcd | 8 mcd | 100 mcd | 9000 mcd | ─ | 14000 mcd |

**REFERENCES**

[1] J. Barber, *Chem. Soc. Rev.* **2009**, *38*, 185.

[2] H. Zhang, W. Tian, L. Zhou, H. Sun, M. Tade, S. Wang, *Appl. Catal. B Environ.* **2018**, *223*, 2.

[3] P. Zhang, Y. Tong, Y. Liu, J. J. M. Vequizo, H. Sun, C. Yang, A. Yamakata, F. Fan, W. Lin, X. Wang, W. Choi, *Angew. Chemie - Int. Ed.* **2020**, *132*, 16343.

[4] F. Liu, Y. Tong, C. Li, X. Liu, *J. Phys. Chem. Lett.* **2021**, *12*, 10359.

[5] Z. Jin, Q. Zhang, J. Chen, S. Huang, L. Hu, Y. J. Zeng, H. Zhang, S. Ruan, T. Ohno, *Appl. Catal. B Environ.* **2018**, *234*, 198.

[6] A. Galushchinskiy, Y. Zou, J. Odutola, P. Nikačević, J.-W. Shi, N. Tkachenko, N. López, P. Farràs, O. Savateev, *Angew. Chem. Int. Ed.* **2023**, *62*, e2023031815.

[7] S. Wang, B. Y. Guan, X. Wang, X. W. D. Lou, *J. Am. Chem. Soc.* **2018**, *140*, 15145.

[8] W. Wang, Z. Wang, Q. Zhu, G. Han, C. Ding, J. Chen, J. R. Shen, C. Li, *Chem. Commun.* **2015**, *51*, 16952.

[9] H. Pang, G. Zhao, G. Liu, H. Zhang, X. Hai, S. Wang, H. Song, J. Ye, *Small* **2018**, *14*, 1.

[10] K. P. Sokol, D. Mersch, V. Hartmann, J. Z. Zhang, M. M. Nowaczyk, M. Rögner, A. Ruff, W. Schuhmann, N. Plumeré, E. Reisner, *Energy Environ. Sci.* **2016**, *9*, 3698.

[11] K. P. Sokol, W. E. Robinson, J. Warnan, N. Kornienko, M. M. Nowaczyk, A. Ruff, J. Z. Zhang, E. Reisner, *Nat. Energy* **2018**, *3*, 944.

[12] V. W. Lau, D. Klose, H. Kasap, F. Podjaski, M. Pignié, E. Reisner, G. Jeschke, B. V. Lotsch, *Angew. Chemie - Int. Ed.* **2017**, *129*, 525.

[13] D. Mersch, C. Y. Lee, J. Z. Zhang, K. Brinkert, J. C. Fontecilla-Camps, A. W. Rutherford, E. Reisner, *J. Am. Chem. Soc.* **2015**, *137*, 8541.

[14] M. Huang, W. Lei, M. Wang, S. Zhao, C. Li, M. Wang, H. Zhu, *J. Mater. Chem. A* **2020**, *8*, 3845.

[15] H. Zhou, T. Liu, X. Zhao, Y. Zhao, H. Lv, S. Fang, X. Wang, F. Zhou, Q. Xu, J. Xu, C. Xiong, Z. Xue, K. Wang, W. C. Cheong, W. Xi, L. Gu, T. Yao, S. Wei, X. Hong, J. Luo, Y. Li, Y. Wu, *Angew. Chemie - Int. Ed.* **2019**, *58*, 18388.

[16] J. Zhong, J. J. Deng, B. H. Mao, T. Xie, X. H. Sun, Z. G. Mou, C. H. Hong, P. Yang, S. D. Wang, *Carbon N. Y.* **2012**, *50*, 335.

[17] P. Cai, X. Feng, J. Fei, G. Li, J. Li, J. Huang, J. Li, *Nanoscale* **2015**, *7*, 10908.

[18] X. Fang, K. P. Sokol, N. Heidary, T. A. Kandiel, J. Z. Zhang, E. Reisner, *Nano Lett.* **2019**, *19*, 1844.

[19] J. Li, X. Feng, Y. Jia, Y. Yang, P. Cai, J. Huang, J. Li, *J. Mater. Chem. A* **2017**, *5*, 19826.

[20] J. Li, J. Li, X. Feng, J. Fei, P. Cai, J. Huang, *J. Mater. Chem. A* **2016**, *4*, 12197.

[21] I. Kim, N. Jo, M. Y. Yang, J. Kim, H. Jun, G. Y. Lee, T. Shin, S. O. Kim, Y. S. Nam, *ACS Appl. Bio Mater.* **2019**, *2*, 2109−2115.

[22] W. Tian, H. Zhang, J. Sibbons, H. Sun, H. Wang, S. Wang, *Adv. Energy Mater.* **2021**, *11*, 2100911.

[23] T. Bobrowski, F. Conzuelo, A. Ruff, V. Hartmann, A. Frank, T. Erichsen, M. M. Nowaczyk, W. Schuhmann, *Chempluschem* **2020**, *85*, 1396.

[24] K. L. Saar, P. Bombelli, D. J. Lea-Smith, T. Call, E. M. Aro, T. Müller, C. J. Howe, T. P. J. Knowles, *Nat. Energy* **2018**, *3*, 75.

[25] M. Sawa, A. Fantuzzi, P. Bombelli, C. J. Howe, K. Hellgardt, P. J. Nixon, *Nat. Commun.* **2017**, *8*, 1.

[26] R. I. Pinhassi, D. Kallmann, G. Saper, H. Dotan, A. Linkov, A. Kay, V. Liveanu, G. Schuster, N. Adir, A. Rothschild, *Nat. Commun.* **2016**, *7*, 1.

[27] P. Bombelli, T. Müller, T. W. Herling, C. J. Howe, T. P. J. Knowles, *Adv. Energy Mater.* **2015**, *5*, 1401299.

[28] N. Sekar, Y. Umasankar, R. P. Ramasamy, *Phys. Chem. Chem. Phys.* **2014**, *16*, 7862.

[29] L. Liu, S. Choi, *J. Power Sources* **2021**, *506*, 230251.

[30] F. L. Ng, S. M. Phang, V. Periasamy, K. Yunus, A. C. Fisher, *Sci. Rep.* **2017**, *7*, 1.

[31] R. W. Bradley, P. Bombelli, D. J. Lea-Smith, C. J. Howe, *Phys. Chem. Chem. Phys.* **2013**, *15*, 13611.

[32] A. E. Inglesby, K. Yunus, A. C. Fisher, *Phys. Chem. Chem. Phys.* **2013**, *15*, 6903.

[33] P. Bombelli, M. Zarrouati, R. J. Thorne, K. Schneider, S. J. L. Rowden, A. Ali, K. Yunus, P. J. Cameron, A. C. Fisher, D. Ian Wilson, C. J. Howe, A. J. McCormick, *Phys. Chem. Chem. Phys.* **2012**, *14*, 12221.

[34] A. J. McCormick, P. Bombelli, A. M. Scott, A. J. Philips, A. G. Smith, A. C. Fisher, C. J. Howe, *Energy Environ. Sci.* **2011**, *4*, 4699.

[35] P. Bombelli, R. W. Bradley, A. M. Scott, A. J. Philips, A. J. McCormick, S. M. Cruz, A. Anderson, K. Yunus, D. S. Bendall, P. J. Cameron, J. M. Davies, A. G. Smith, C. J. Howe, A. C. Fisher, *Energy Environ. Sci.* **2011**, *4*, 4690.
